# Rapid Turnover of Long Noncoding RNAs and the Evolution of Gene Expression

**DOI:** 10.1371/journal.pgen.1002841

**Published:** 2012-07-26

**Authors:** Claudia Kutter, Stephen Watt, Klara Stefflova, Michael D. Wilson, Angela Goncalves, Chris P. Ponting, Duncan T. Odom, Ana C. Marques

**Affiliations:** 1 Cancer Research UK, Cambridge Research Institute, Li Ka Shing Centre, Cambridge, United Kingdom; 2 University of Cambridge, Cambridge, United Kingdom; 3 European Bioinformatics Institute, European Molecular Biology Laboratory, Hinxton, United Kingdom; 4 Wellcome Trust Sanger Institute, Hinxton, United Kingdom; 5 MRC Functional Genomics Unit, Department of Physiology, Anatomy and Genetics, University of Oxford, Oxford, United Kingdom; 6 Department of Physiology, Anatomy, and Genetics, University of Oxford, Oxford, United Kingdom; Massachusetts Institute of Technology, United States of America

## Abstract

A large proportion of functional sequence within mammalian genomes falls outside protein-coding exons and can be transcribed into long RNAs. However, the roles in mammalian biology of long noncoding RNA (lncRNA) are not well understood. Few lncRNAs have experimentally determined roles, with some of these being lineage-specific. Determining the extent by which transcription of lncRNA loci is retained or lost across multiple evolutionary lineages is essential if we are to understand their contribution to mammalian biology and to lineage-specific traits. Here, we experimentally investigated the conservation of lncRNA expression among closely related rodent species, allowing the evolution of DNA sequence to be uncoupled from evolution of transcript expression. We generated total RNA (RNAseq) and H3K4me3-bound (ChIPseq) DNA data, and combined both to construct catalogues of transcripts expressed in the adult liver of *Mus musculus domesticus* (C57BL/6J), *Mus musculus castaneus*, and *Rattus norvegicus*. We estimated the rate of transcriptional turnover of lncRNAs and investigated the effects of their lineage-specific birth or death. LncRNA transcription showed considerably greater gain and loss during rodent evolution, compared with protein-coding genes. Nucleotide substitution rates were found to mirror the *in vivo* transcriptional conservation of intergenic lncRNAs between rodents: only the sequences of noncoding loci with conserved transcription were constrained. Finally, we found that lineage-specific intergenic lncRNAs appear to be associated with modestly elevated expression of genomically neighbouring protein-coding genes. Our findings show that nearly half of intergenic lncRNA loci have been gained or lost since the last common ancestor of mouse and rat, and they predict that such rapid transcriptional turnover contributes to the evolution of tissue- and lineage-specific gene expression.

## Author Summary

The best-understood portion of mammalian genomes contains genes transcribed into RNAs, which are subsequently translated into proteins. These genes are generally under high selective pressure and deeply conserved between species. Recent publications have revealed novel classes of genes, which are also transcribed into RNA but are not subsequently translated into proteins. One such novel class are long noncoding RNA (lncRNA). LncRNA loci are controlled in a similar manner to protein-coding genes, yet are more often expressed tissue-specifically, and their conservation and function(s) are mostly unknown. Previous reports suggest that lncRNAs can affect the expression of nearby protein-coding genes or act at a distance to control broader biological processes. Also, lncRNA sequence is poorly conserved between mammals compared with protein-coding genes, but how rapidly their transcription evolves, particularly between closely related species, remains unknown. By comparing lncRNA expression between homologous tissues in two species of mouse and in rat, we discovered that lncRNA genes are “born” or “die” more rapidly than protein-coding genes and that this rapid evolution impacts the expression levels of nearby coding genes. This local regulation of gene expression reveals a functional role for the rapid evolution of lncRNAs, which may contribute to biological differences between species.

## Introduction

The mammalian transcriptome has recently been shown to be surprisingly diverse in its extent and encoded functions [1]–[3], much of which are noncoding RNAs (ncRNAs) as they are not translated into proteins. The ability to sequence the entire transcriptome in an unbiased manner has not only allowed more complete characterization of well described and highly abundant noncoding RNAs with known function, such as transfer RNAs, small nuclear RNAs, small nucleolar RNAs and ribosomal RNAs, but have also revealed additional ncRNA species. For example, a number of long ncRNAs (lncRNAs) larger than 200 nucleotides (nt) have been discovered [2], [4], [5]. Many lncRNA loci are intergenic, when transcription occurs wholly within the genomic intervals between two adjacent protein-coding genes [6]. Some lncRNAs can be transcribed divergently from a neighbouring protein-coding transcript using identical or almost identical transcriptional initiation complexes [6]. In addition, lncRNAs overlapping with protein-coding genes can be transcribed from either strand [6]–[8].

Although the precise roles of many lncRNAs remain unknown, in general they are thought to act in transcriptional regulation [6], [9], [10]. LncRNAs can regulate gene expression programs through a variety of mechanisms, including interactions with chromatin remodelling complexes or transcription factors [11]. Consistent with a *cis*-regulatory role, co-expression of intergenic lncRNA loci with their neighbouring protein-coding genes has been observed [12], [13] and a number of intergenic lncRNAs have demonstrated roles in regulating the expression of genes in their genomic vicinity [9]. Some intergenic lncRNAs appear to regulate the expression of both neighbouring and distal genes [14], [15]. Indeed, many intergenic lncRNAs have been experimentally demonstrated to have roles in regulating transcription of distally located targets, in *trans* [16]. Nevertheless, the exact proportion and the distinguishing features of *cis*- and *trans*-acting intergenic lncRNAs remain unknown.

If lncRNAs' functional roles are conserved it is expected that their loci should be evolutionarily preserved. Indeed, the transcripts and promoters of mammalian intergenic lncRNAs exhibit signatures of selective constraint: their promoters are highly conserved across vertebrates [2] and they have accumulated fewer substitutions than neighbouring putative neutral sequence [17], [18]. However little is yet known of the evolutionary persistence of lncRNA transcription. Generally the loss and gain of functional noncoding sequence can occur rapidly, with approximately half of all functional ancestral nucleotides predicted to have been gained or lost in mouse or rat since their common ancestor [19]. Other noncoding RNAs, in particular tRNAs, have been shown to exhibit rapid turnover of their transcribed loci, despite conservation of their function [20]. Turnover of regulatory elements underlies species-specific transcriptional evolution and may be associated with phenotypic changes [21].

Only a small minority of intergenic lncRNAs in mouse or human were found to have transcribed orthologous sequences in the other species [22], [23]. This might reflect turnover of transcribed loci, or it might imply that intergenic lncRNAs, which are often lowly expressed and tissue specific [6], [9], [18], [23], have transcribed orthologous sequences that remain undetected. Indeed, analysis of the transcription of three intergenic lncRNA loci across homologous regions of the mammalian and avian brain revealed that some intergenic lncRNAs can have conserved expression patterns [24].

To resolve the extent of lncRNA transcriptional turnover it is important to undertake a careful comparison of lncRNA transcription in homogeneous and homologous tissues. Achieving this in closely related species also allows the distinction of transcriptional turnover from DNA sequence turnover and furthermore might reveal otherwise unexpected mechanisms of regulatory divergence. Here we experimentally and computationally explored the genetic structure and function of lncRNA loci in matched tissues from three closely related rodent species, *Mus musculus domesticus* (C57BL/6J), *Mus musculus castaneus* and *Rattus norvegicus*.

## Results

### Combining RNAseq and chromatin status to identify long noncoding RNAs in mouse liver

**Figure 1:**
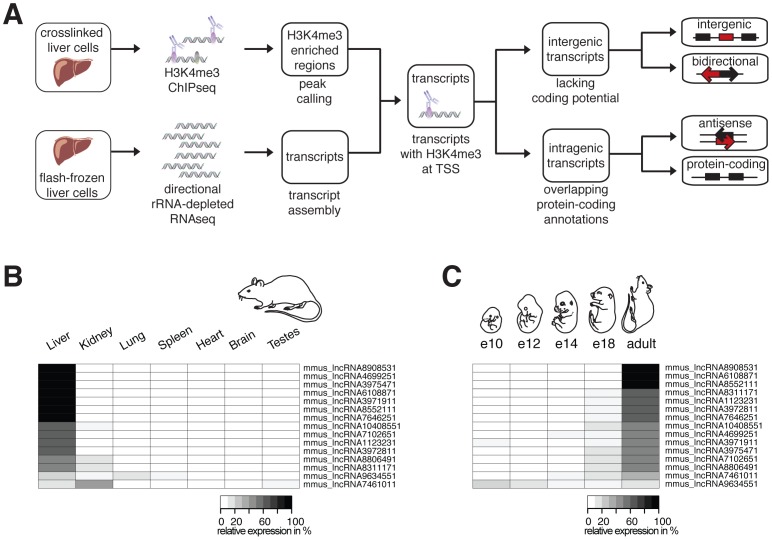
Identification and characterization of ncRNAs in Mmus. (A) Primary tissues were isolated and separate portions either flash frozen to permit RNA sequencing or treated with formaldehyde to crosslink protein-DNA contacts, which allows the chromatin immunoprecipitation reaction. Flow diagram illustrates assembly of liver expressed transcripts (RNAseq) marked by H3K4me3 at their transcriptional start sites (TSSs). Classification of long noncoding RNA (lncRNA, red) based on genomic location relative to annotated protein-coding genes (black) and directionality of transcription (arrows). Relative expression of 15 randomly selected intergenic lncRNA transcripts in (B) seven different adult Mmus tissues and (C) at five different developmental stages of Mmus liver was validated by RT-qPCR. Each heatmap row represents a single intergenic lncRNA. Areas are shaded according to the relative level of transcription in different tissues and developmental stages (in percent white: 0 to black: 100%).

We identified transcripts expressed in the liver of three young adult male *Mus musculus domesticus* (inbred strain C57BL/6J termed hereafter Mmus) individuals by directional, stranded ribosomal RNA (rRNA)-depleted transcriptome sequencing (total RNAseq) (Figure 1A) (see Materials and Methods). Data from three independent biological replicates were pooled. About 80% of sequencing reads were mapped [25] to the reference Mmus (mm9) genome and liver gene expression was detectable for 61% of all UTRs and coding exons annotated in the mouse genome (coverage: 66%). We found that a substantial fraction of sequencing reads map to unannotated, likely noncoding, loci consistent with previous results [26].

Using our total transcriptome sequencing data we assembled *de novo* 56917 transcripts [27] expressed in the Mmus liver (Figure 1A). As a consequence of the short-read single end nature of our data, our transcripts can be fragmented due to incomplete coverage of the full-length cDNA. To identify independent transcripts, we performed genome-wide chromatin immunoprecipitation followed by sequencing (ChIPseq) against trimethylation of lysine 4 of histone H3 (H3K4me3), which marks the beginning of actively transcribed genes [28] and identified enriched regions [29] (Figure 1A) (see Materials and Methods).

We intersected the genomic locations of 18303 H3K4me3 enriched regions with the predicted 5′ end of our RNAseq-defined Mmus transcripts longer than 200 bases in length, thereby predicting 8915 distinct transcription start sites (TSSs) (Figure 1A). As found in previous studies, we identified a limited number of protein-coding genes that exhibited evidence of bidirectional transcription at their TSS (Figure S1, Table S10) [30]. Most of these transcribed regions are likely noncoding and are not further addressed in our study except when supported by a *de novo* assembled noncoding transcript [31].

Similarly, we identified transcripts that were either intergenic (*n* = 388) or intragenic (*n* = 8527) based on their overlap with Mmus protein-coding gene annotations (Figure 1A) (see Materials and Methods). Intergenic transcripts lacking protein-coding potential [32] were annotated as long intergenic ncRNAs (intergenic lncRNAs) (*n* = 316, Table S3). Next we defined transcribed loci as clusters of one or more transcripts with overlapping exonic or intronic nucleotides. From 293 of these loci only intergenic lncRNA transcripts were expressed (Table S3 and S4). The vast majority (*n* = 233) of these intergenic lncRNA loci have no overlap with intergenic lncRNAs annotated in the mouse genome by Ensembl (build 64), demonstrating that current mouse intergenic lncRNA catalogues are largely incomplete [18]. Mmus liver intergenic lncRNAs transcripts were significantly (two-tailed Mann-Whitney test, typically *p*<1×10^−4^) found to be: (i) more lowly expressed, (ii) shorter and (iii) to have fewer exons than their protein-coding transcript counterparts (Table S2) consistent with previous reports [23], [33].

The second group of 7289 intragenic loci comprises 8527 transcripts overlapping protein-coding genes (Ensembl build 60, Table S3 and S4). Forty-nine loci have overlapping antisense RNAs transcribed from the opposite strand and marked by separate H3K4me3 enriched regions indicating independent transcriptional initiation (Table S9). Examples in this category include the constitutively expressed noncoding RNA *Kcnq1ot1* [34].

### lncRNAs show spatio-temporal expression patterns in mouse

Most protein-coding genes are expressed in multiple tissues [35]. In contrast, lncRNA expression tends to be spatially and temporally restricted [6], [18], [23], [36]. We validated the expression of 15 randomly selected liver expressed intergenic lncRNA transcripts by quantitative PCR (RT-qPCR) in seven Mmus adult tissues (Figure 1B) and nine intragenic antisense lncRNA transcripts by strand specific RT-qPCR [8] in four adult tissues (Figure S2D). These tissues were chosen because they show different degrees of cell type complexity and biological functionality. We found that the large majority of the tested intergenic and intragenic antisense lncRNA transcripts are predominately expressed in liver.

Large changes in gene expression are observed during tissue development [37]. In order to identify whether the intergenic lncRNAs we identified are developmentally regulated during hepatocyte differentiation, we measured the abundance of representative lncRNAs by RT-qPCR at embryonic stages E10, E12, E14 and E18 and adult stage P62. Our data showed that lncRNAs are also extremely specific to the adult developmental stage of liver. In summary, the intergenic lncRNAs we identify are specifically expressed in nutritionally unstressed adult liver (Figure 1C).

### Collection of matched long noncoding RNAs in castaneus and rat

**Figure 2:**
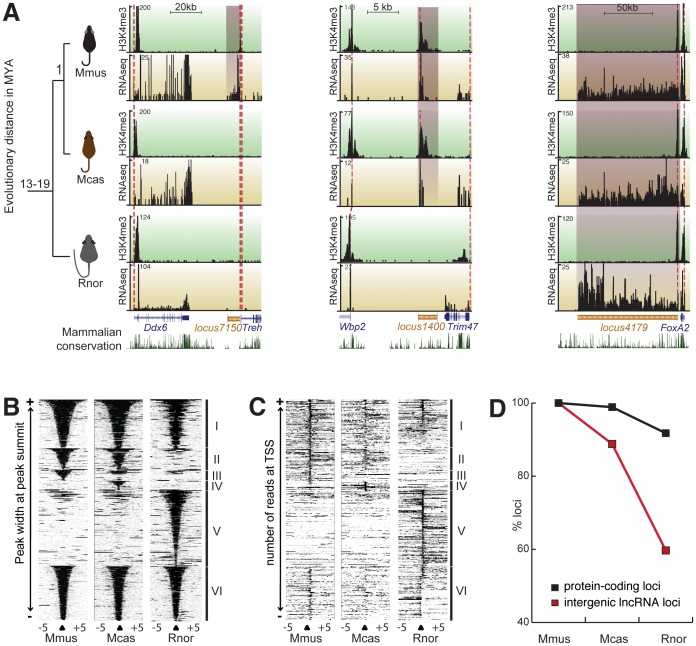
Transcriptional turnover of liver expressed transcripts in rodents. (A) Primary liver tissue was isolated from Mmus and two other rodents whose lineage split from Mmus one million years (Mcas) or 13 to 19 million years (Rnor). Examples of a Mmus-specific (*locus7150*, left), *Mus* genus-conserved (*locus1400*, middle), and rodent conserved (*locus4179*, right) lncRNA locus and their corresponding neighbouring protein-coding genes are illustrated. H3K4me3 enrichment is shown against a green background track and RNAseq signature against a yellow background track. The height (y-axis) of each track corresponds to the read depth. Beneath the enrichment tracks is the Refseq genome annotation for this region (UCSC genome browser). The mammalian conservation track (UCSC genome browser) shows degree of placental mammal base pair conservation (20 species) and sequence conservation. The syntenic positions of the predicted TSS of ncRNAs and neighbouring protein-coding loci are traced between species with dashed red lines. (B) H3K4me3 enriched regions (black: H3K4me3 bound DNA reads and white: no ChIPseq reads) within 5 kb of the peak summit for all identified intergenic lncRNA (one per line, categories I to V) and 136 randomly sampled protein-coding loci (category VI). Categories represent intergenic lncRNA loci that are transcribed and marked by H3K4me3 in all three rodents (I), in Mmus and Mcas but not in Rnor (II), in Mmus only (III), in Mcas only (IV) and Rnor only (V). Peaks were sorted according to their width. (C) Heatmap similar to (D) representing intergenic lncRNA transcripts anchored on predicted TSS (black: more than one RNAseq reads and white: less than one RNAseq reads). (D) Transcriptional turnover of liver-expressed protein coding (black) or intergenic lncRNA loci (red) in rodents.

Sequence comparison of mouse intergenic lncRNAs and their human and rat orthologous sequence have shown that these transcripts tend to be constrained, an evolutionary hallmark of functionality, albeit at much lower levels than protein-coding genes [17], [18]. However little is yet known about transcriptional turnover of lncRNA during evolution. To address the transcriptional turnover of lncRNAs, we explored their transcription across three rodents. In addition to Mmus, we studied transcript expression in the adult liver of a closely related mouse *Mus musculus castaneus* (CAST/EiJ termed Mcas) and in the rat (*Rattus norvegicus*, termed Rnor) (Figure 2). The two mouse subspecies, Mmus and Mcas, diverged about one million years ago (MYA) and last shared a common ancestor with Rnor about 13 to 19 MYA [38] (Figure 2A). These differences in species separation across evolutionary time allowed us to take two snapshots of transcriptional turnover during rodent evolution, using the closest wild-derived mouse species (Mcas) to Mmus that is commercially available and Rnor as the evolutionary nearest rodent species with a well-annotated genome. Similar to the characterization of transcripts in Mmus liver, we performed RNAseq and H3K4me3 ChIPseq experiments in Mcas and Rnor, and identified 158 and 605 intergenic lncRNAs respectively (Tables S1, S5, S6, S7, S8). The observed difference between the numbers of annotated intergenic lncRNA loci across the three rodents (293, 158 and 605 for Mmus, Mcas and Rnor, respectively) can be either due to experimental bias or underlying biology. To test the contribution of the difference in read number of each species RNAseq library (Table S1), we reassembled transcripts in Mmus and Rnor after randomly selecting from Mmus and Rnor libraries the same number of reads as Mcas, our smallest library (Table S1). For each species we repeated this procedure 10 times. By comparing the numbers of intergenic lncRNAs in Mmus or Rnor that overlapped a transcript from these recreated libraries, we found that the differences in numbers of lncRNAs between mice (Mmus and Mcas) species are mostly due to the depth of sequencing. After adjusting the read number of the Mmus RNAseq library to the Mcas RNAseq library, we identified a mean of 154 intergenic lncRNA loci (standard deviation = 3.4) for Mmus, a similar number to the one assembled in Mcas (n = 158), suggesting that the difference in the number of lncRNA loci is due to an experimental bias. In contrast, in Rnor, using the same number of sequencing reads the reduction approach afforded a mean of 284 intergenic lncRNA loci (standard deviation = 5.9). This number corresponds to a 80% rise over the 158 Mcas intergenic lncRNA loci and indicates that there is an increase of liver lncRNA loci in the rat lineage.

### Rapid turnover of lncRNA transcription

We next considered if during rodent evolution lncRNA loci were conserved in their transcription in a similar manner to protein-coding genes. We defined transcriptional turnover as instances of genomic loci for which syntenic sequence is conserved between two or more species yet transcription of this conserved sequence is not. To determine conservation of transcribed loci, we combined H3K4me3 peaks with RNA sequencing reads overlapping (by more than 1 bp) the syntenic regions to create a stringent set of conserved loci (see Materials and Methods). These loci show evidence of both transcriptional initiation and transcript formation. Owing to the availability of its larger number of publicly available genome wide resources, such as spatial and temporal expression patterns [39], we anchored our analysis on Mmus. To allow differentiation between sequence and transcriptional turnover we only considered Mmus loci that have aligned orthologous sequence in the rat genome [intergenic lncRNA loci *n* = 268 (91.5%), protein-coding loci *n* = 6723 (92.2%)].

We then classified mouse loci according to their transcriptional conservation into three classes: those specific to Mmus, if evidence of expression was found only in Mmus; those conserved in *Mus* genus, when evidence of transcription was found in Mmus and Mcas but not in Rnor; and, those conserved across these rodents, when expression evidence was found in Mmus, Mcas and Rnor (Figure 2A, Table S4). Our definition does not explicitly take into account conservation of exon-intron structure. Globally, H3K4me3 and RNAseq signals were grouped according to our classification (Figure 2B–2C).

In order to confirm that the observed differences were not solely a consequence of biases introduced by sequencing depth, we validated our interspecies comparisons by semi-quantitative RT-PCR in independent biological replicates from adult livers of Mmus, Mcas and Rnor for 24 intergenic lncRNA transcripts from four categories (rodent conserved, *Mus* genus conserved, Mmus-specific, and Rnor-specific, Figure S3). These RT-PCR results confirmed that our global approach accurately identifies species- and lineage-specific intergenic lncRNAs.

Turnover of transcription is considerably more frequent for intergenic lncRNA loci than for protein-coding genes in the rodent liver (Figure 2D). A significantly smaller fraction of intergenic lncRNA than protein-coding loci exhibit conserved transcription across rodents [intergenic lncRNA loci *n* = 160 (59.7%), protein-coding loci *n* = 6169 (91.7%), two-tailed Fisher's exact test, *p*<10^−3^]. Conversely, a significantly higher proportion of intergenic lncRNA than protein-coding loci are specific to the Mmus lineage [intergenic lncRNA loci *n* = 30 (11.2%), protein-coding loci *n* = 75 (1.1%), two-tailed Fisher's exact test, *p*<10^−3^].

The difference in sequencing depth between the three species influenced the number of annotated intergenic lncRNAs. To account for this effect and provide a more conservative estimate of transcriptional conservation we considered the set of intragenic and lncRNA loci that were assembled after adjusting the Mmus and Rnor RNAseq library sizes to that of Mcas (see Materials and Methods). Intragenic and intergenic lncRNA loci were annotated as previously. We considered a Mmus locus to have conserved expression if it had an overlapping H3K4me3 peak and an overlapping transcript (>1 bp). As previously, we found protein-coding gene loci to be more often conserved in rodents (1326/2415, 55%) than intergenic lncRNA loci (31/110, 28%, two-tailed Fisher's exact test, *p*<10^−3^).

Next we aimed to gain initial insights into the conservation of exon-intron structures of Mmus intergenic lncRNAs. For mouse intergenic lncRNAs and protein-coding loci whose transcription was conserved in rat (160 and 6169 loci, respectively) we compared the coverage by RNAseq reads of mouse exonic nucleotides in the rat orthologous regions. We found that rodent conserved protein-coding transcripts have a significantly higher coverage (median 78%) than intergenic lncRNA (median 47%, two-tailed Mann-Whitney test, *p*<2×10^−16^, Figure S4). This observation can be a consequence of lower coverage of low abundance transcripts and/or lower conservation of exon-intron structure for intergenic lncRNAs.

Similarly, we observed that the transcriptional conservation of noncoding transcripts that overlap protein-coding genes in antisense orientation also showed a rapid decay across rodent evolution. Only 36% of the Mus conserved intragenic antisense transcripts are expressed in Rnor (Figure S2). These results indicate that the large majority of ncRNAs are conserved in the *Mus* genus but not in the evolutionarily further distant species Rnor. The apparent low conservation of intragenic antisense transcription is consistent with previous conservation analysis [33].

To investigate transcriptional turnover of intergenic lncRNAs beyond the rodent lineage, we used publicly available polyA^+^ transcriptome sequencing data for the adult human liver (Human BodyMap 2.0 RNAseq data). Rodents and human shared a common ancestor over 90 MYA [40]. We considered in this analysis only Mmus transcripts whose expression was supported by at least one overlapping polyA^+^ sequencing read [41]. We found that the majority of mouse intergenic lncRNA loci overlap polyA^+^ reads (273/293 loci), suggesting that few intergenic lncRNA loci assembled here transcribe only non-polyadenylated transcripts. We discarded 1368 (18.8%) protein-coding and 159 (58.2%) intergenic lncRNA loci in Mmus that lack an apparent orthologous sequence in the human or rat genome [42]. As observed for the rodent lineage, a significantly smaller fraction of Mmus intergenic lncRNA than protein-coding genes orthologous in humans are expressed in the liver [intergenic lncRNA loci (*n* = 76, 56.7%), protein-coding loci (*n* = 5689, 96.1%), two-tailed Fisher's exact test, *p*<10^−3^) (Figure S5). Our data indicate that the fraction of liver transcribed mouse intergenic lncRNAs expressed in the orthologous region of the human genome is two-fold higher (two-tailed Fisher's exact test, *p*<10^−3^) than prior estimates [22], which supports the use of homologous tissue types to investigate levels of transcriptional conservation of tissue specific transcripts, such as intergenic lncRNAs. We conclude that rapid turnover of intergenic lncRNAs is not restricted to the rodent lineage, but is widespread among eutherian mammals.

### Sequence constraint is associated with conservation of intergenic lncRNA transcription

Next we examined how sequence constraint reflects transcriptional conservation of intergenic lncRNA and protein-coding loci. For each transcript we considered its most 5′ nucleotide to correspond to the transcriptional start site and defined its promoter as the 400 nucleotides upstream of this site. We compared the mouse-rat nucleotide substitution rate for intergenic lncRNA loci (*d_loci_*) and promoters (*d_promoter_*), to rates for genomically neighbouring and non-overlapping ancestral repeats [ARs (*d_AR_*)] with matched G+C content [18], [43]. ARs are transposable element-derived sequences that were present in the last common ancestor of human and mouse; most of these sequences have been observed to evolve neutrally and hence provide reliable proxies for local neutral mutation rates [44]. We first confirmed that Mmus liver-expressed intergenic lncRNA loci accumulated mutations at a significantly slower rate than adjacent neutral sequence (Figure S6A) (*d_loci_* = 0.148, *d_AR_* = 0.164, two-tailed Mann-Whitney test, *p*<3×10^−7^). In line with this observation, long sequence segments that have preferentially purged insertions or deletions in Mmus and Rnor lineages were 1.6-fold enriched in intergenic lncRNA transcription over expected levels (permutation test, *p*<10^−3^) [44]. As previously reported [12], [18] the sequences of intergenic lncRNA loci evolve more rapidly than those of full-length protein-coding loci (Figure S6B) (*d_loci_*/*d_AR_* = 0.902; protein-coding *d_loci_*/*d_AR_* = 0.857; two-tailed Mann-Whitney test, *p*<2×10^−3^). Additionally, the putative core promoters of intergenic lncRNAs accumulated significantly more substitutions than those of protein-coding genes (Figure S6C) (intergenic lncRNA *d_promoter_*/*d_AR_* = 0.843; protein-coding *d_promoter_*/*d_AR_* = 0.746, two-tailed Mann-Whitney test, *p*<2×10^−5^). The discrepancy between this result and published findings [2] is likely due to the incompleteness of lncRNA transcripts' 5′ ends and thus to incomplete delineation of lncRNA promoter sequences.

**Figure 3:**
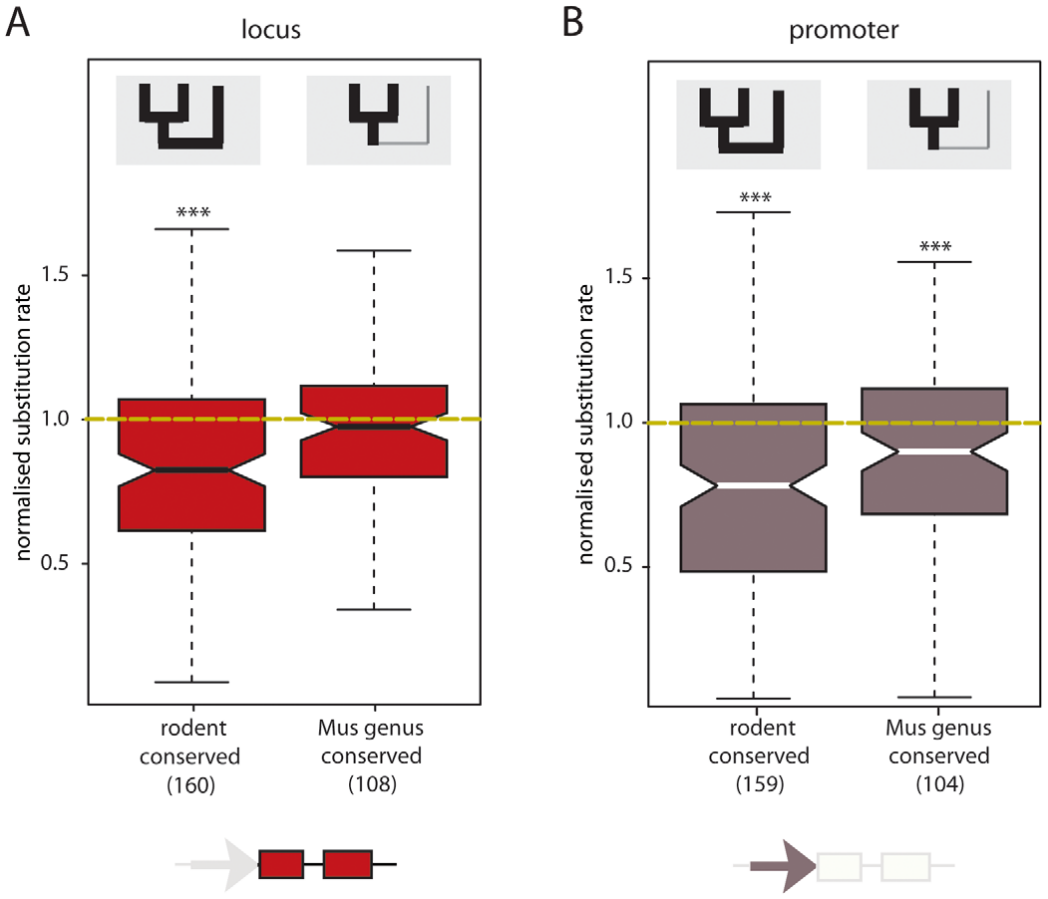
Rodent conserved intergenic lncRNA loci and promoter sequences exhibit constraint. Normalised nucleotide substitution rates for (A) 160 intergenic lncRNA loci conserved in rodents (expressed in Mmus, Mcas and Rnor) and 108 *Mus* genus specific intergenic lncRNA loci; and (B) 159 putative intergenic lncRNA promoters conserved in rodents and 104 *Mus* genus specific intergenic lncRNA putative promoters. Putative proximal intergenic lncRNA promoters were defined as the 400 bp upstream region of the predicted TSS. Yellow dashed line represents the expected neutral substitution rate. Compared to neutral sequence (ancestral repeats, AR) in the vicinity, nucleotide substitution rates differ significantly for loci and promoter of intergenic lncRNA transcripts conserved in rodents (as indicated by asterisks ***, *p*<0.001) and the promoters of *Mus* genus specific intergenic lncRNAs (***, *p*<0.001).

To determine whether loss of transcription is associated with loss of sequence constraint, we compared Mmus to Rnor nucleotide substitution rates between two groups of intergenic lncRNAs: those specific to the *Mus* genus (Mmus and Mcas) and those conserved among these rodents (Mmus, Mcas and Rnor). Rodent conserved intergenic lncRNA loci show evidence for purifying selection on both transcribed (two-tailed Mann-Whitney test, *p*<4×10^−10^) (Figure 3A) and putative promoter sequences (two-tailed Mann-Whitney test, *p*<3×10^−12^) (Figure 3B). Intergenic lncRNA loci transcribed in the *Mus* genus but not in Rnor, exhibit no constraint in transcribed regions (two-tailed Mann-Whitney test, *p*>0.2) (Figure 3A). *Mus* genus-conserved putative core promoters accumulated significantly fewer substitutions than neighbouring putatively neutral sequence (median *d_prom_* = 0.151 and *d_AR_* = 0.165, two-tailed Mann-Whitney test, *p*<5×10^−3^) suggesting they evolved under purifying selection (Figure 3B). Negative selective pressure was significantly higher on the promoters of loci with rodent conserved transcription than on promoter sequence with *Mus* genus-specific transcription (rodent conserved median *d_prom_*/*d_AR_* = 0.783, *Mus* genus-specific median *d_prom_*/*d_AR_* = 0.901, two-tailed Mann-Whitney test, *p*<7×10^−3^).

We asked whether the observed low degree of sequence constraint on intergenic lncRNA loci, relative to protein-coding genes, was due to rapid transcriptional turnover of a subset of intergenic lncRNAs. To test this, we compared Mmus to Rnor nucleotide substitution rates for the transcribed sequences (including exons and introns) between the subset of intergenic lncRNA loci exhibiting conserved expression in the rodent liver (*n* = 160) with the corresponding set of protein-coding genes (*n* = 6641) and found no significant difference (intergenic lncRNA *d_loci_*/*d_AR_* = 0.827, protein-coding *d_loci_*/*d_AR_* = 0.842 two-tailed Mann-Whitney test, *p*>0.58) (Figure S7A). For loci conserved in rodents, nucleotide substitution rates of intronic and exonic sequence were compared between Mmus and Rnor. Introns (*d_intron_*) of protein-coding genes and intergenic lncRNAs evolved at comparable rates (intergenic lncRNA *d_intron_*/*d_AR_* = 0.959, protein-coding *d_intron_*/*d_AR_* = 0.986, two-tailed Mann-Whitney test, *p*>0.28) (Figure S7C). In contrast, protein-coding gene exons evolve under strong purifying selection (intergenic lncRNA *d_exon_*/*d_AR_* = 0.805, protein-coding *d_exon_*/*d_AR_* = 0.484, two-tailed Mann-Whitney test, *p*<10^−15^) (Figure S7B) likely to ensure the maintenance of their coding potential during evolution.

Our results therefore indicate that intergenic lncRNA loci that were gained or lost in recent *Mus* evolution evolved neutrally between mouse and rat. Conversely, rodent conserved intergenic lncRNAs have accumulated fewer substitutions than neighbouring neutral sequence indicating that conservation of transcription is reflected in sequence constraint.

### Intergenic lncRNA loci tend to lie adjacent to protein-coding genes with liver function

Mammalian intergenic lncRNA loci and their genomically adjacent protein-coding genes show a significant tendency to exhibit similar spatiotemporal expression profiles [12], [13], [15], [23], [45]. We found intergenic lncRNA transcription in liver occurs significantly more frequently near to protein-coding genes that are expressed in the liver [39] than expected by chance (see Materials and Methods; 1.6-fold; permutation test, *p*<5×10^−3^). Complementary results were obtained using Database for Annotation, Visualization, and Integrated Discovery (DAVID) tissue annotation categories (Figure S8) [46]. About 30% of the protein-coding genes closer to intergenic lncRNA loci were classified as liver expressed (*p*<3×10^−5^).

### Lineage-specific intergenic lncRNA expression is associated with increased transcription of adjacent protein-coding genes

We considered whether lineage-specific transcription of intergenic lncRNAs might associate with the expression level of genomically adjacent protein-coding genes (see Materials and Methods). If intergenic lncRNAs have no effect on nearby protein-coding gene expression, then lineage-specific differences in gene expression of genes should be unaffected by whether a neighbouring intergenic lncRNA locus is transcribed.

The existence of relatively large numbers of lineage-specific intergenic lncRNAs in mouse and rat permitted this hypothesis to be tested using Mmus and Rnor. Two additional reasons that we specifically analysed the intergenic lncRNAs identified in these two species were (i) the high quality of the genome annotations, relative to Mcas, and (ii) the existence of other published datasets that permitted further validation of our results [20].

First, we normalised gene expression for Mmus and Rnor RNAseq data (see Materials and Methods, Figure S9A) and validated the fold-difference on 17 selected protein-coding mRNA by RT-qPCR (Figure S9C and S9D). In order to obtain a baseline for transcriptional variation between species from this normalised set, we first estimated the fold difference in liver expression between 230 Mmus housekeeping protein-coding genes [47] and their one-to-one orthologous genes in Rnor (median fold-difference in expression = 0.020, see Materials and Methods). Next, we identified the closest protein-coding gene for each conserved or lineage-specific Mmus or Rnor intergenic lncRNA. We selected the intergenic lncRNA loci whose neighbouring protein-coding genes had annotated [48] one-to-one orthologs in the second species (Table S9).

**Figure 4:**
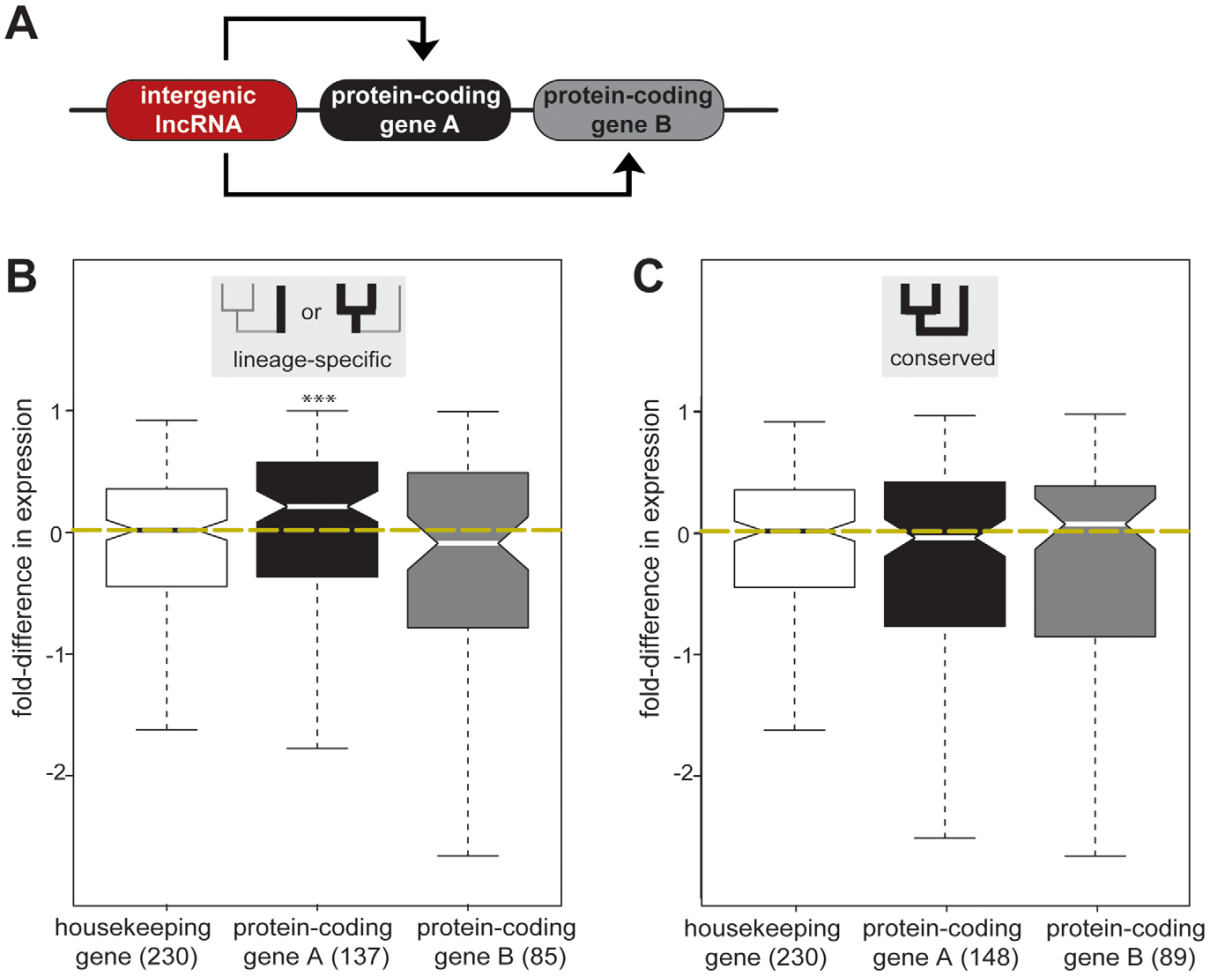
Lineage-specific intergenic lncRNAs are associated with increased expression of genomically adjacent protein-coding genes. (A) Effect of intergenic lncRNA (red) transcription on their closest protein-coding genes A (black) and their respective closest protein-coding genes B (grey) was determined. (B) Fold-difference in expression for one-to-one orthologous protein-coding gene pairs (A and B) where gene A is adjacent to lineage-specific (Mus genus- or Rnor-specific) intergenic lncRNA loci. The fold-difference in expression between Mmus and Rnor for protein-coding gene A is significantly (represented by asterisks [**], *p*<0.005) higher than the expected variation in expression based on 230 housekeeping genes (white). Yellow dashed line represents median fold-difference in expression between Mmus and Rnor housekeeping genes. Lineage-specific intergenic lncRNA transcription has no significant effect on the expression levels of protein-coding genes B. (C) Fold-difference in expression for one-to-one orthologous protein-coding gene pairs (A and B) where genes A are adjacent to rodent conserved (conserved in Mmus, Mcas and Rnor) intergenic lncRNAs loci. Rodent conserved intergenic lncRNA gene expression has no significant effect on the transcription of neighbouring protein-coding gene A or B between mouse and rat. In parentheses are the numbers of protein-coding genes studied.

We found that the expression levels of the genes whose nearest intergenic lncRNA locus showed conserved expression between rodents (*n* = 148) were similar to housekeeping gene levels (median fold-difference = −0.035, two-tailed Mann-Whitney test, *p*>0.36) (Figure 4, Table S12).

We then asked whether gene expression levels alter when a nearby intergenic lncRNA is gained or lost in one species. In contrast to the conserved situation above, we found that those protein-coding genes A nearest to lineage-specific intergenic lncRNA loci (*n* = 137) tended to be expressed at a higher level, with a median increase in gene expression of approximately 25% (median fold-difference = 0.212, two-tailed Mann-Whitney test, *p*<0.005) (Figure 4, Table S12). We repeated this analysis and confirmed this result using an independent dataset [20]. We found that the median expression levels of protein-coding gene loci adjacent to lineage-specific intergenic lncRNA loci were significantly higher than those of protein-coding gene loci near conserved intergenic lncRNA loci (two-tailed Mann-Whitney test, *p*<7×10^−5^) (Figure S9B, Figure S10). Transcription increased for half (50%) of those protein-coding genes lying adjacent to lineage-specific intergenic lncRNA loci, when assessed using either total RNA or mRNA expression; in contrast, less than a third (29%) of protein-coding genes near conserved intergenic lncRNA loci show consistent increased expression in both datasets (two tailed Fisher's exact test, *p*<0.05, Figure S11), suggesting that in some cases gain or loss of intergenic lncRNAs may influence the expression levels of neighbouring genes. We next investigated if some relative orientations of lineage-specific lncRNA transcription were more frequently associated with increased expression of the most proximal protein-coding gene. We divided lineage-specific intergenic lncRNA and protein-coding gene pairs into three classes (Figure S12A): tandem (48 gene pairs) if transcription occurred in the same orientation, divergent (71 gene pairs), or convergent (17 gene pairs) if transcription occurred in opposite directions either diverging or converging, respectively. All three relative genomic arrangements are associated with increased expression of the closest protein-coding genes. Both tandem and convergent orientations are associated with significantly increased expression at the 5% level while divergent orientation is significant at the 10% level (*p*<0.08, Figure S12B).

We considered a number of possible interpretations for this apparent association of lineage-specific intergenic lncRNAs with increased transcription of nearby protein-coding genes. The increased gene expression could be either (i) due to regional modifications to the genome that co-ordinately influence all coding and noncoding loci [49] or (ii) correlated with the transcription of the proximal intergenic lncRNA locus [13], [15]. A key distinguishing feature between these two mechanisms is whether lineage-specific expression of intergenic lncRNAs is associated with regional increases in transcription.

To test this, we identified the next most proximal protein-coding gene B, beyond its closest protein-coding gene A (Figure 4A). Genes duplicated in tandem often share regulatory elements and, as a consequence, exhibit similar expression patterns [50]. To account for this evolutionary bias, we excluded 17 protein-coding genes B that were annotated [48] as protein-coding gene A paralogs (see Materials and Methods). In contrast to the observed lineage-specific effects on protein-coding genes A, the expression levels of protein-coding genes B were not significantly affected (two-tailed Mann-Whitney test, *p*>0.7) by either conserved (median fold-difference = 0.078) or lineage-specific (median fold-difference = −0.088) intergenic lncRNA transcription (Figure 4, Table S13).

We next tested whether similar results might be obtained for lineage-specific protein-coding genes. We used the previously identified set of *Mus*-genus lineage-specific expressed protein-coding genes. We identified genes A′ as the closest protein-coding genes to these loci, protein-coding A′ (Figure S13). We excluded paralogous protein-coding gene pairs and considered only protein-coding genes A′ with a one-to-one ortholog in rat (89 genes). Transcription levels of nearby genes appear unaffected by the presence of lineage-specific protein-coding gene transcription in the genomic vicinity (median fold-difference = 0.052, two-tailed Mann-Whitney test, *p*>0.4) (Figure S13). As an additional control, we compared the densities of chromatin boundary elements (CCCTC-binding factor [CTCF]-bound sites) and DNase I hypersensitivity sites in the intergenic regions between (i) the lineage-specifically expressed intergenic lncRNA locus and its neighboring protein-coding gene A and (ii) protein-coding genes B, using data from previous studies [51], [52]. We found no significant differences between these densities (permutation test *p*>0.2). The association between lineage-specific lncRNA transcription and increased expression levels of neighbouring protein-coding genes might depend on the distance between their transcriptional start sites (TSSs). The median distance of the TSS of a lineage-specifically expressed intergenic lncRNA with its closest protein-coding gene is 22 kb. However, no significant correlation was observed between this distance and the median fold difference in expression for protein-coding genes measured between mouse and rat (Pearson correlation, R = −0.03, *p* = 0.76, Figure S14).

Our comparison of matched tissues in two species thus revealed that birth or death of intergenic lncRNAs is associated with changes in transcription of proximal protein-coding genes.

## Discussion

To investigate the evolution of lncRNAs, we identified the highest confidence set of lncRNAs in matched, nutritionally unstressed, adult livers of three closely related rodent species: Mmus, Mcas and Rnor, by combining genome-wide interrogation of chromatin signatures and total RNA expression. This highly conservative set of lncRNAs confirmed a number of prior observations. First, many intergenic and antisense lncRNA loci are expressed in a cell/tissue- or time-specific manner: we found that the intergenic lncRNAs present in adult liver are not only absent from other adult tissues, but are perhaps surprisingly even absent in developing mouse liver. These temporally- and spatially- restricted expression patterns, together with their relatively low expression levels, likely explain why our intergenic lncRNA set shows limited overlap with previously reported sets [18]. From our analysis, two major results emerged: first, that intergenic and antisense lncRNA transcription can evolve extremely rapidly between closely related mammals; second, that this rapid evolution seems to occur simultaneously with increased expression of neighbouring protein-coding genes.

### Evolution of intergenic and antisense lncRNA transcription between closely related mammals

Previous studies have indicated that 12 to 15% of lncRNAs are conserved between human and mouse, based on comparison of EST and cDNA datasets from disparate experimental designs [22], [23]. Our matched interspecies data are perhaps better suited to establish experimentally the rate of lncRNA turnover. The use of mouse and rat, being closely related species, minimises the effects of genomic sequence divergence, thus better uncoupling sequence and transcriptional changes. Transcription of noncoding loci is more frequently gained or lost than transcription of protein-coding genes; between 28% and 61% of intergenic and antisense lncRNAs, respectively are specific to the *Mus* genus. We expect similar turnover will be found in most cell types of various developmental stages given that liver is a typical somatic tissue [53]. The transience of intergenic lncRNA transcription is mirrored by changes to selective pressures acting on their sequences. Our results are consistent with purifying selection acting on transcribed intergenic lncRNA loci, and with no selection acting on untranscribed orthologous sequence in other species. This coupling of transcriptional conservation with sequence constraint suggests that conserved intergenic lncRNA loci are biologically significant in rodents.

### Lineage-specific intergenic lncRNAs and transcription of neighbouring protein-coding genes

The expression levels of intergenic lncRNAs and their genomically neighbouring protein-coding genes have previously been shown to be positively correlated [12], [13]. We find that species-specific transcription of intergenic lncRNAs correlates with elevated expression of neighbouring protein-coding genes. The increased transcription observed among neighbouring genes is unique to intergenic lncRNAs, and seems unlikely to be due to local changes in chromatin environment. If the intergenic lncRNAs in other tissues and species behave similarly, intergenic lncRNAs could contribute substantially to lineage-specific and tissue-specific evolution of gene expression.

The rapid turnover we observed in lncRNA transcription strongly resembles what was recently reported for transcription factor binding events [54]–[56], tRNA transcription [20] and functional regulatory sequences in general [19]. For instance, between 10 to 20% of transcription factor binding events overlap between human and mouse liver [56], which is similar in scale to what we now find for intergenic lncRNAs. These parallels suggest that rapid evolution is a general feature of noncoding regulatory mechanisms.

It was recently proposed that intergenic lncRNAs have minimal impact on the transcriptional regulation of their neighbouring protein-coding genes [16], [23]. By exploiting the rapid birth and death of noncoding RNAs, we revealed that intergenic lncRNAs could contribute to lineage-specific changes in the expression levels of neighbouring protein-coding genes. Our data do not preclude distal regulatory roles, which might be lineage-specific, for some or all intergenic lncRNAs we investigate. It will now be crucial to understand how intergenic lncRNAs evolve and to unravel the molecular mechanisms underlying lineage-specific gene expression changes associated with intergenic lncRNAs.

## Materials and Methods

### Tissue preparation

ChIPseq, RNAseq, and RT-PCR experiments were performed on liver material isolated from three rodents: *Mus musculus domesticus* (Mmus), *Mus musculus castaneus* (Mcas), and *Rattus norvegicus* (Rnor). Each ChIPseq and RNAseq experiments were performed on at least two independent biological replicates from different animals. Mmus and Mcas (male adults, 10 weeks old) were obtained from the Cambridge Research Institute. Rnor (male adults, 9 weeks old) were obtained from Charles River. All tissues were either treated post-mortem with 1% formaldehyde for ChIP experiments or flash-frozen in liquid N_2_ for RNA experiments. The investigation was approved by the ethics committee and followed the Cambridge Research Institute guidelines for the use of animals in experimental studies under Home Office license PPL 80/2197.

### Library and sequencing preparation

ChIP sequencing experiments were performed as described previously [57] using H3K4me3 antibody (CMA304) [58]. In brief, the immunoprecipitated DNA was end-repaired, A-tailed, ligated to the sequencing adapters, amplified by 18 cycles of PCR and size selected (200–300 bp). For RNA-sequencing library preparation, total RNA was extracted using Qiazol reagents (Qiagen) and DNase-treated (Turbo DNase, Ambion). Ribosomal RNA was depleted from total RNA using RiboMinus (Invitrogen). RNA was reversed transcribed and converted into double-stranded cDNA (SuperScript cDNA synthesis kit, Invitrogen), sheared by sonication followed by paired end adapter (Illumina) ligation and prior to PCR amplification cDNA was UNG-treated to maintain strand-specificity [59]. After passing quality control on a Bioanalyzer 1000 DNA chip (Agilent) libraries were sequenced on the Illumina Genome Analyzer II (single-ended) and post-processed using the standard GA pipeline software v1.4 (Illumina).

### Read mapping and next-generation sequencing (NGS) data analysis

H3K4me3 ChIPseq and associated input DNA control ChIPseq reads were aligned to the corresponding reference genomes (mm9 for *Mus musculus domesticus* and *Mus musculus castaneus*; Rn4 for *Rattus norvegicus*) using MAQ version 0.7.1 (default parameters) [60]. Reads mapping to multiple genomic locations were discarded. Genomic regions enriched over matching input DNA control were defined using MACS version 1.3.7.1 using the default parameters [61]. Comparative analysis was carried out using the Galaxy web tool [62]. Total RNA sequencing reads were mapped with Tophat (version 1.3.0) [25], using default parameters. A file containing the mapped coordinates of mouse and rat ESTs and mRNA mapped coordinates (downloaded from UCSC on the 11^th^ March 2011) was provided to facilitate total RNA read mapping across splice junction for Mmus and Mcas, and Rnor respectively. Reads mapping to rRNA, tRNA and mtRNA were masked and the remainder were used to assemble transcripts *de novo* using Cufflinks (version 1.3.0) [27].

### Transcript and promoter annotation

We filtered out transcripts smaller than 200 nucleotides (nt) and without an H3K4me3 peak overlapping their predicted transcriptional start site (TSS). Transcripts overlapping protein-coding gene annotations (by one or more base pair) from RefSeq, Ensembl (build 60) [48] and UCSC were annotated as intragenic. To discriminate between unannotated protein-coding and putatively noncoding transcripts we estimated the coding potential of all intergenic transcripts using the coding potential calculator (CPC) [32]. We annotated all transcripts with a coding potential less than 0 as intergenic long noncoding RNAs (intergenic lncRNAs). The 400 nt region upstream of the 5′ end (TSS) of each intergenic lncRNA or protein-coding transcript was annotated as a putative promoter. Transcribed loci were defined as non-overlapping regions with one or more transcripts that can contain overlapping exonic or intronic nucleotides. Loci containing only transcripts predicted to be intergenic lncRNAs were annotated as intergenic lncRNA loci. The remainder were annotated as protein-coding loci.

For the identification of antisense transcripts from the Cufflinks output file (n = 56917), we first identified 2383 transcripts overlapping protein-coding genes in antisense orientation in Mmus. This number included four types of ambiguous cases that were systematically removed: (i) annotated protein-coding transcripts (removing 1816 transcripts), (ii) antisense transcripts lacking an H3K4me3 peak independent from the TSS of overlapping protein-coding gene (removing 324 transcripts), (iii) transcripts lacking H3K4me3 marks at their 5′ end, and (iv) mapping assembly artefacts, revealed by visual inspection (collectively removing 90 transcripts). Taking all of these cases into consideration, 49 loci (or 153 antisense transcripts) were annotated in Mmus. A similar procedure was conducted in Mcas and Rnor, revealing 66 loci in total.

To identify lncRNAs deriving from bidirectional transcription at TSSs of protein-coding genes, we subtracted divergently transcribed protein-coding genes from our list of actively transcribed protein-coding genes. The TSSs of gene loci are spanned by one H3K4me3 peak and the evidence of divergent transcription is represented by RNAseq reads mapping in opposite directions. We identified divergent reads within an 1 kb window of a protein-coding gene's annotated TSS (Ensembl, build 60) [30].

Heatmaps and transcription start site aggregation plots were constructed using seqMINER [63].

To account for the difference in RNAseq library size between the three rodent species (Table S1) Mmus and Rnor transcripts were assembled using the same number of reads in Mcas library, the smallest RNAseq library. Reads were randomly selected without replacement and transcripts reassembles using Cufflinks and annotated as described above.

### Reverse transcription and quantitative PCR (RT-qPCR)

RT-PCR analysis of lncRNAs was performed by reverse transcription of 10 µg of DNase-treated total RNA according to the manufacturer's protocols using 200 U SuperScript-II Reverse Transcriptase (Invitrogen Corporation), 0.5 µg oligo(dT) and 0.5 µg random primers or 1 µg gene-specific primers (see Table S11). Negative controls were included in RT reactions. The cDNAs were then treated with RNase H at 37°C for 1 hour. Each PCR reaction typically contained 25 ng of cDNA, 5 pmol of the gene-specific primers (Table S11), 10 µL PCR Master Mix (Bioline), and 2 µL of the diluted cDNAs in a total volume of 20 µL. Reactions were carried out in triplicate in ABI 7900HT Fast Real-Time PCR system at the optimal temperature, as defined by provider instructions.

### Genome-wide associations

The significance of genome-wide associations between intergenic lncRNAs and their neighbouring protein-coding genes was assessed using Genome Association Tool (GAT) (Heger *et al.*, in preparation). GAT compares the observed number of overlapping nucleotides between a set of segments with particular annotations to what would be expected from random placement of these segments. Expected densities are obtained using a randomisation procedure that accounts for G+C content and chromosome specific biases. A previous version of GAT was used in [9], [18]. This tool infers associations between intergenic lncRNA loci (segments) across the following annotation sets: (I) mouse-to-rat indel purified segments [44] and (II) liver-expressed protein-coding gene territories (Average Difference values >200) [39]. A protein-coding gene territory is defined as the genomic region containing all nucleotides that are closer to the gene than they are to its most proximal up- and downstream protein-coding genes, as described elsewhere [9], [18]. As a second tool, we used the gene functional classification tool Database for Annotation, Visualization, and Integrated Discovery (DAVID) (default parameters: count = 2 and ease = 0.1) [46] to explore the enrichment of tissue gene expression.

Regions of the mouse and rat genome that are enriched in CTFC binding were obtained from [51]. DNase hypersensity sites (DHS) in the mouse adult liver were obtained from [52]. Only male and sex independent DHS peaks that were either annotated as being robust and standard were considered in this analysis. GAT (Heger et al., in preparation) was used to test the observed density of these two class of regulatory elements in the intergenic region between lineage-specific intergenic lncRNAs and protein coding gene A (Figure 4) to what would be expected based on their distribution across the intergenic regions between lineage-specific intergenic lncRNA and protein-coding gene B (Figure 4).

### Transcriptional conservation

Orthologous regions between Mmus and Rnor were identified using whole genome pairwise alignments [42]. An intergenic lncRNA locus was considered to be expressed in another species when its orthologous (between *Mus* species and Rnor) or equivalent (between Mmus and Mcas) position had an overlapping (>1 bp) H3K4me3 peak and one or more overlapping RNAseq reads. Due to the lack of H3K4me3 data for human, overlap (>1 bp) by one or more RNAseq reads in the orthologous human location was considered sufficient evidence for transcriptional conservation of an Mmus locus in human sequence. Only Mmus loci whose transcription was supported by one or more polyA^+^ selected sequencing read [41] were considered in this analysis. Identical criteria were used to determine the conservation of antisense lncRNA loci. An antisense lncRNA locus was judged to be expressed in another species when its orthologous position had an overlapping (>1 bp) H3K4me3 peak and one or more overlapping RNAseq reads in opposite orientations. We visually inspected these calls on 66 loci across the three rodent species.

### Nucleotide constraint

Nucleotide constraint between Mmus and Rnor locus, exon, intron or putative promoter was estimated as described previously [18]. Pairwise substitution rates between Mmus and Rnor genomic regions were estimated using BASEML from the PAML package with the REV substitution model [64]. The substitution rate of the region of interest was compared to the rate observed for non-overlapping adjacent (<500 kb) ancestral repeats (inserted before the primate and rodent split) with similar G+C content [18].

### Gene expression

Mmus and Rnor protein-coding transcript annotations were downloaded from Ensembl (build 60, http://www.ensembl.org/index.html) and used to define a set of constitutive exons for each gene. To account for differences in size of constitutively expressed portions of Mmus and Rnor genes, the total number of overlapping reads per nucleotide in Rnor was adjusted to what would be expected if the sequence in Rnor had the same length as that observed in Mmus. The expression of a gene in Rnor or Mmus is proportional to the sum of reads mapped to their exons divided by their combined length. To allow comparison of gene expression between species, read counts were normalized using TMM (edgeR package) [65]. Briefly, to estimate the normalised library size for each species, it was assumed that 60% of expressed genes were transcribed at similar levels in the two species. Other cut-offs (50% and 70%) yielded similar results. The normalised Mmus and Rnor library size was used to calculate the expression level (as total number of fragments per kb of sequence per million reads mapped, FPKM) of each gene in each species.

### Gene expression differences between mouse and rat

Each intergenic lncRNA locus was paired with its genomically closest protein-coding gene. Only pairs whose protein-coding genes had one-to-one orthologs between Mmus and Rnor were considered. The fold difference in expression levels of protein-coding genes associated with lineage-specific (Mus-genus or Rnor-specific) or rodent conserved expression was estimated between [6] the same direction. To calculate the fold difference in expression for each housekeeping gene between Mmus and Rnor species *X* and *Y* were randomly assigned. Fold expression differences for protein-coding genes B or A′ (Figure 4, Figure S12) were calculated in a similar manner.

### Statistical analysis

Apart from permutation tests all other statistical analysis were performed using the R package [66].

### Accession code

RNAseq and H3K4me3 ChIPseq sequencing data are available from ArrayExpress under accession number E-MTAB-867. Additional mRNAseq data used was E-MTAB-424.

## Supporting Information

Figure S1 Noncoding RNA transcription at or near Mmus bidirectional promoters. (A) Representative genome browser view of a bidirectional promoter. *Entpd8* gene is expressed in Mmus liver and exhibits transcription in antisense orientation on the complementary strand that is supported by several sequencing reads. H3K4me3 enrichment is shown with green background track and RNAseq reads with yellow background track, color-coded red for reads on the reverse strand and black on the forward strand relative to Entpd8. The y-axis of each track represents read number. Beneath is the genome annotation of this region obtained from RefSeq (UCSC browser) with arrows indicating the direction of transcription. The mammalian conservation track (UCSC genome browser) shows degree of placental mammal base pair conservation (20 species). (B) The aggregation plot displays the mean coverage of RNAseq reads of 378 Ensembl genes (black, forward strand) with evidence of RNA transcription in close proximity on the reverse strand (red) in a 5 kb region centred at the start of transcription (TSS). (C) Aggregation plot (as in B) representing the mean coverage of RNAseq signals of 200 randomly selected liver-expressed protein-coding genes (red, forward strand).
(TIF)

Figure S2 Validation of intragenic antisense ncRNA transcripts in rodents. (A) *lncRNA-530* is located in antisense orientation to *Acmsd* and *lncRNA-530* expression is conserved in Mmus, Mcas, and Rnor. H3K4me3 enrichment is shown with green background track and RNAseq signatures with yellow background track, color-coded blue for l*ncRNA-530* located on the reverse strand and pink for *Acmsd* located on the forward strand. The y-axis of each track represents read number. Beneath is the genome annotation in this region obtained from RefSeq (UCSC browser) with arrows indicating the direction of transcription. The mammalian conservation track (UCSC genome browser) shows degree of placental mammal base pair conservation (20 species) and sequence conservation. (B) Represents *lncRNA 441*, which is located in antisense orientation to *Per2* and only present in the Mus genus. (C) Shows the rat-specific *lncRNA 6503*, which is located in antisense orientation to *Adcy1*. (D) Transcript abundance of selected intragenic antisense lncRNAs in different adult Mmus tissues was validated by strand-specific quantitative RT-PCR. Each heatmap row represents one lncRNA. Areas are shaded according to abundance in per cent (white: 0 to black: 100%). (E) A three-way VENN diagram representing the intersect between the intragenic antisense lncRNA genes identified in each of the three rodents used in this study. Areas are shaded according to number of lncRNA genes (white: low to black: high).Validation of intragenic antisense ncRNA transcripts in rodents.
(TIF)

Figure S3 Validation of intergenic lncRNA expression in rodents. Liver expression of selected intergenic lncRNAs in Mmus, Mcas, and Rnor was tested by RT-PCR amplification: (A) Mmus, Mcas, and Rnor conserved intergenic lncRNAs, (B) Mus-genus specific intergenic lncRNAs, (C) Mmus-specific intergenic lncRNAs and (D) Rnor-specific intergenic lncRNAs. *Actin B* (*ActB*) expression in the three species was used as RT-PCR control. Genomic DNA (gDNA) of Rnor (B) and Mmus (D) was used for validating RT-PCR result.
(TIF)

Figure S4 RNAseq read coverage is significantly higher accross protein-coding exons compared to intergenic lncRNAs. Coverage of rat RNAseq reads on rodent conserved intergenic lncRNA exons and protein-coding exons was determined. Exons of protein-coding transcrips have significantly higher coverage than those of intergenic lncRNAs (as indicated by asterisks ***, p<0.001). In parentheses are the numbers of intergenic lncRNA and protein-coding transcripts studied.
(TIF)

Figure S5 Transcriptional turnover of liver expressed intergenic lncRNA and protein-coding gene loci between rodents and human. (A) Phylogenetic tree representing the evolutionary relationship between human (Hsap), rat (Rnor) and mouse (Mmus). Humans and rodents shared a common ancestor about 80 to 90 million years ago (MYA). (B) Transcriptional turnover of liver-expressed Mmus protein-coding (black) and intergenic lncRNA loci (red) between rodents and human.
(TIF)

Figure S6 Nucleotide constraint between mouse and rat for promoter and transcribed loci of Mmus expressed intergenic lncRNAs and protein-coding genes. The cumulative distributions of substitution rates between mouse and rat for (A) 279 Mmus intergenic lncRNA loci (red) and neighbouring (<500 kb) ancestral repeat loci (AR, blue). Median substitution rate for intergenic lncRNA (*d_loci_* = 0.148) and AR (*d_AR_* = 0.164) indicates that intergenic lncRNA loci accumulated significantly fewer substitutions than ARs (two-tailed Mann-Whitney test, *p*<3×10^−7^). (B) 279 Mmus-expressed intergenic lncRNA loci (red) and 7040 Mmus protein-coding genes (black). Intergenic lncRNA loci (median *d_loci_*/*d_AR_* = 0.902) in comparison to protein-coding transcripts (median *d_loci_*/*d_AR_* = 0.857) accumulated substitutions at significantly higher rates (two-tailed Mann-Whitney test, *p*<2×10^−3^). (C) 276 Mmus intergenic lncRNA (red) and 6921 Mmus protein-coding proximal putative promoters (black). Putative proximal promoters are defined as the 400 bp upstream regions of the TSS. Between mouse and rat the proximal promoters of intergenic lncRNA evolved faster (median *d_promoter_*/*d_AR_* = 0.843) than those of protein-coding genes (median *d_promoter_*/*d_AR_* = 0.746) (two-tailed Mann-Whitney test, *p*<2×10^−5^). The substitution rate for each loci were normalised to the substitution rate measured for AR with matched G+C content in their vicinity (<500 kb). Numbers of loci studied are shown in parentheses. Black dashed line indicates 50% of the cumulative proportion.
(TIF)

Figure S7 Nucleotide constraint between mouse and rat for different transcript features of rodent conserved intergenic lncRNA loci and protein-coding genes. The cumulative distributions of substitution rates between mouse and rat is shown for (A) 160 intergenic lncRNA loci and 6641 protein-coding gene loci whose expression is conserved between mouse and rat. Median substitution rates for intergenic lncRNA loci (*d_loci_*/*d_AR_* = 0.827) and protein-coding genes (*d_loci_*/*d_AR_* = 0.842) are not significantly different (two-tailed Mann-Whitney test, *p*>0.58). (B) Cumulative distribution of substitution rates for the exonic sequence of 160 intergenic lncRNA loci and 6641 protein-coding loci whose expression is rodent conserved. Intergenic lncRNA exons evolved under significantly (Mann-Whitney test, *p*<10^−15^) less constraint (median *d_exon_*/*d_AR_* = 0.805) than those of protein-coding genes (median *d_exon_*/*d_AR_* = 0.484). (C) Cumulative distribution of substitution rates for the intronic sequence of 26 intergenic lncRNA loci and 4571 protein-coding genes. Intergenic lncRNA introns (median *d_intron_*/*d_AR_* = 0.959) accumulated substitutions at similar rates to introns in protein-coding genes (median *d_intron_*/*d_AR_* = 0.986) (two-tailed Mann-Whitney test, *p*>0.28). The substitution rate for each loci were normalised to the substitution rate measured for ancestral repeats (AR) with similar G+C content in their vicinity (<500 kb). Cumulative proportion plots for intergenic lncRNAs and protein-coding genes are represented in red and in black, respectively. Number of loci studied are shown in parentheses. Black dashed line indicates 50% of the cumulative proportion.
(TIF)

Figure S8 Expressed protein-coding genes located near intergenic lncRNAs hold liver-associated functional annotation. Classification of functional annotation (tissue) of protein-coding genes near intergenic lncRNAs. Left: percentage of protein-coding genes with assigned functional annotation (black bars), middle: functional annotation and right: false discovery rate (green bars). Each row represents one functional annotation (tissue).
(TIF)

Figure S9 Normalised expression values of Mmus and Rnor one-to-one orthologous protein-coding genes correlate. Normalised expression level estimates [log(FPKM)] based on (A) total RNA and (B) mRNA sequencing reads of mouse (x-axis) and rat (y-axis) are positively correlated. Pearson correlation (R) are reported at bottom right of each panel. Median fold differences (log scale) of selected protein-coding gene products obtained from (C) RNAseq experiments were validated by (D) RT-qPCR of three independent biological replicates. The order of transcripts selected in (C) was maintained in (D). Yellow: mRNAs upregulated in mouse (or downregulated in rat), grey: transcripts with similar expression fold changes in mouse and rat, and blue: mRNAs downregulated in mouse (or upregulated in rat).
(TIF)

Figure S10 Lineage-specific intergenic lncRNAs are associated with increased expression of genomically adjacent protein-coding genes. mRNAseq based fold-difference in expression for one-to-one orthologous closest protein-coding gene to rodent conserved (conserved in Mmus, Mcas and Rnor) and lineage-specific (Mus genus- or Rnor-specific) expressed intergenic lncRNAs. Rodent conserved intergenic lncRNA gene expression is not associated with increased expression level (median fold-difference in expression = −0.248) of neighbouring protein-coding gene (housekeeping genes median fold difference in expression = 0.051, two-tailed Mann-Whitney test, p>0.18). In contrast lineage specific intergenic lncRNA expression is associated with significantly (2-tailed Mann-Whitney test, *p*<7×10^−5^, represented by asterisks [***]) increased expression of their neighbouring protein-coding genes (median fold difference in expression = 0.455). Yellow dashed line represents median fold-difference in expression between 231 Mmus and Rnor housekeeping genes. Numbers of loci studied are shown in parentheses. Normalised expression values were obtained from mRNAseq experiments [18].
(TIF)

Figure S11 Lineage specific intergenic lncRNA transcription associates with consistent increased expression levels of neighbouring protein-coding genes. Expression levels for most protein-coding genes near lineage-specific intergenic lncRNAs (Mus genus- or Rnor-specific, left) are increased in comparison to protein-coding genes near rodent conserved intergenic lncRNAs (right). White: decrease, grey: ambiguous (inconsistent direction of change between two experiments), black: increase.
(TIF)

Figure S12 Lineage-specific intergenic lncRNAs are associated with increased expression of genomically adjacent protein-coding genes independent of relative orientations. (A) Relative orientations of lineage-specific intergenic lncRNAs (red) and their closest protein-coding neighbouring genes (black) are illustrated. Intergenic lncRNAs are placed downstream of its protein-coding loci in this diagram for illustrative purposes. Intergenic lncRNA and protein-coding gene pairs were divided into three classes: (i) tandem if transcription occurs in the same directions (48 pairs); (ii) convergent (17 pairs); and (iii) divergent (71 pairs). (B) Fold-difference in expression for one-to-one orthologous protein-coding gene pairs adjacent to lineage-specific (Mus genus- or Rnor-specific) intergenic lncRNA loci. The fold-difference in expression for Mmus and Rnor protein-coding genes was higher (two-tailed Mann-Whitney test; tandem: p-value<0.05; convergent: p<0.05; divergent: p<0.1) than the expected variation in expression based on 230 housekeeping genes (white). Yellow dashed line represents median fold-difference in expression between Mmus and Rnor housekeeping genes. In parentheses are the numbers of protein-coding genes studied.
(TIF)

Figure S13 Rodent conserved and lineage-specific protein-coding genes are not associated with elevated expression of their closest neighbouring protein-coding gene. Effect of protein-coding gene (grey) transcription on their closest protein-coding genes A′ (black) was determined for (A) rodent conserved and (B) lineage-specific protein-coding gene pairs. (C) The median fold-difference in expression level between mouse and rat for protein-coding genes A′ closest to protein-coding genes with conserved expression in rodents (median fold-difference = 0.04) is not significantly different from the median fold-difference in expression for 230 housekeeping genes (two-tailed Mann-Whitney test, *p*>0.8). Lineage-specifically expressed protein-coding genes (Mus genus) are also not associated with significant (two-tailed Mann-Whitney test, *p*>0.4) differences in expression of their closest protein-coding gene (median fold-difference = 0.05). The yellow dashed line represents median fold-difference in expression between Mmus and Rnor housekeeping genes. Numbers of protein-coding genes studied are shown in parentheses.
(TIF)

Figure S14 Distance of protein-coding genes to nearest lineage–specific intergenic lncRNA loci and expression levels of protein-coding genes do not correlate. The lineage-specific effect of intergenic lncRNA expression on its neighbouring protein-coding genes does not correlate with the distance between the two loci. The smallest distance (base pairs) between the TSSs for each intergenic lncRNA and protein-coding gene A pair (y-axis, log-scale) is plotted against the corresponding fold-difference in expression between Mmus and Rnor (x-axis, log-scale). These measures are not significantly (*p* = 0.76) correlated (Pearson correlation coefficient R = −0.026).
(TIF)

Table S1 Assembly statistics.
(XLS)

Table S2 Comparison of mouse intergenic lncRNA and protein-coding transcript.
(XLS)

Table S3 Mmus identified transcripts (gff).
(TXT)

Table S4 Mmus transcribed loci.
(TXT)

Table S5 Mcas identified transcripts (gff).
(TXT)

Table S6 Mcas transcribed loci.
(TXT)

Table S7 Rnor identified transcripts (gff).
(TXT)

Table S8 Rnor transcribed loci.
(TXT)

Table S9 Rodent identified antisense transcripts.
(XLS)

Table S10 Mmus identified genes with divergent transcription.
(TXT)

Table S11 List of PCR primers used in this study.
(XLS)

Table S12 Effect of intergenic lncRNA transcription on protein-coding gene A.
(XLS)

Table S13 Effect of intergenic lncRNA transcription on protein-coding gene B.
(XLS)
