# Supplementary figures and images for: Rapid Turnover of Long Noncoding RNAs and the Evolution of Gene Expression
Source: PLoS Genet. 2012 Jul 26;8(7):e1002841. doi: 10.1371/journal.pgen.1002841 (PMC3406015; doi:10.1371/journal.pgen.1002841)

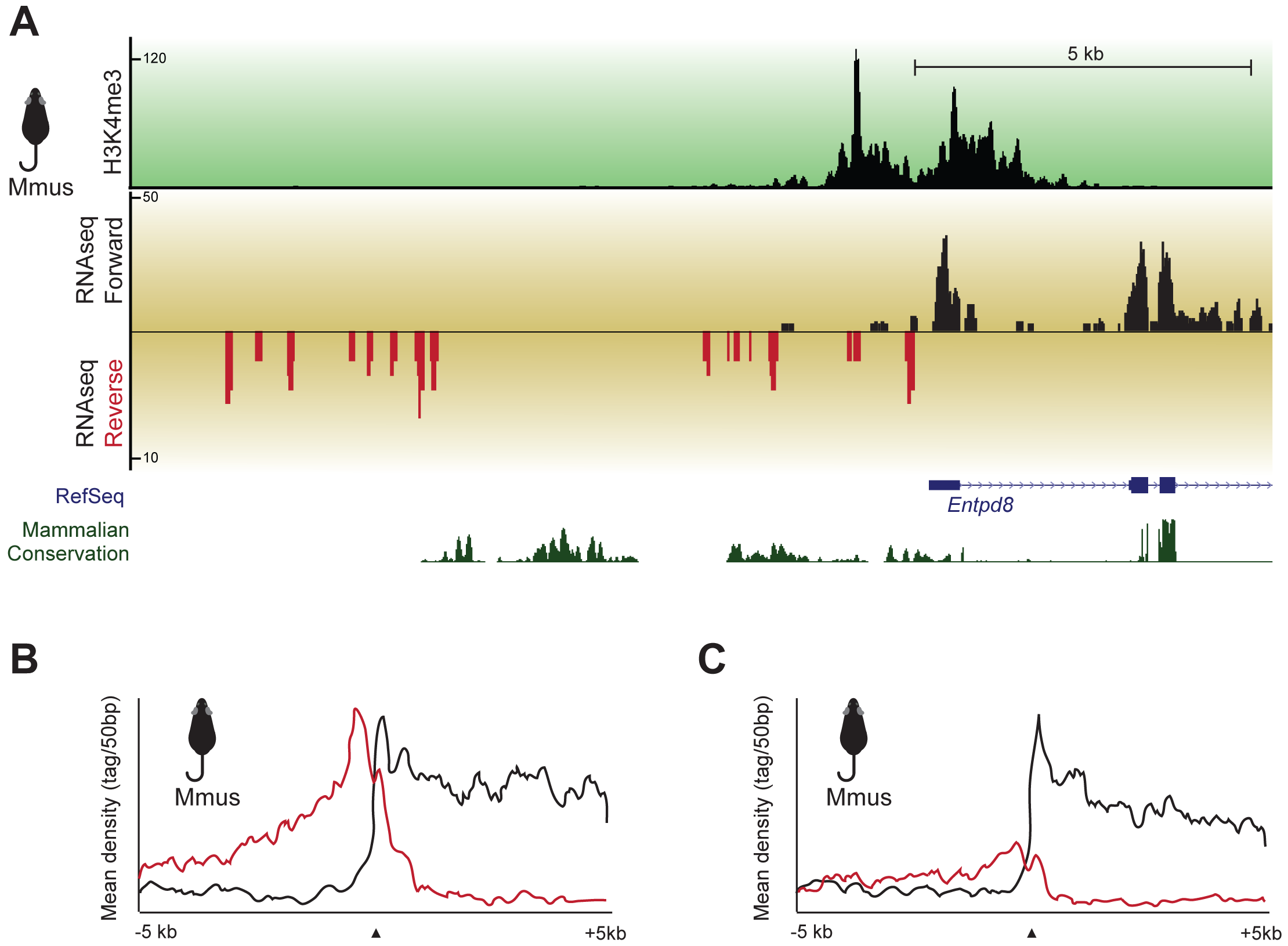

Supplement: Figure S1 — Noncoding RNA transcription at or near Mmus bidirectional promoters. (A) Representative genome browser view of a bidirectional promoter. Entpd8 gene is expressed in Mmus liver and exhibits transcription in antisense orientation on the complementary strand that is supported by several sequencing reads. H3K4me3 enrichment is shown with green background track and RNAseq reads with yellow background track, color-coded red for reads on the reverse strand and black on the forward strand relative to Entpd8. The y-axis of each track represents read number. Beneath is the genome annotation of this region obtained from RefSeq (UCSC browser) with arrows indicating the direction of transcription. The mammalian conservation track (UCSC genome browser) shows degree of placental mammal base pair conservation (20 species). (B) The aggregation plot displays the mean coverage of RNAseq reads of 378 Ensembl genes (black, forward strand) with evidence of RNA transcription in close proximity on the reverse strand (red) in a 5 kb region centred at the start of transcription (TSS). (C) Aggregation plot (as in B) representing the mean coverage of RNAseq signals of 200 randomly selected liver-expressed protein-coding genes (red, forward strand). (TIF) [file pgen.1002841.s001.tif]

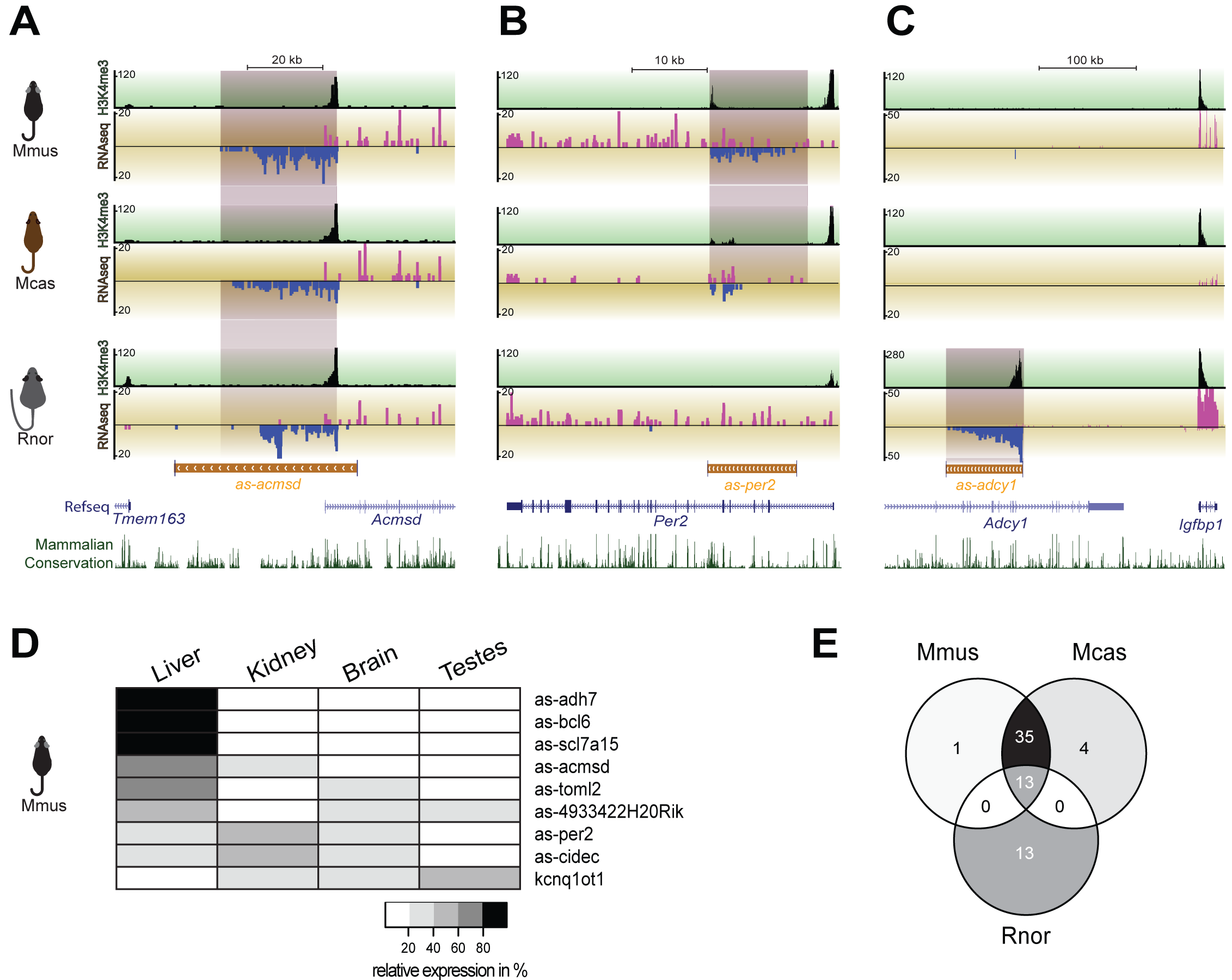

Supplement: Figure S2 — Validation of intragenic antisense ncRNA transcripts in rodents. (A) lncRNA-530 is located in antisense orientation to Acmsd and lncRNA-530 expression is conserved in Mmus, Mcas, and Rnor. H3K4me3 enrichment is shown with green background track and RNAseq signatures with yellow background track, color-coded blue for lncRNA-530 located on the reverse strand and pink for Acmsd located on the forward strand. The y-axis of each track represents read number. Beneath is the genome annotation in this region obtained from RefSeq (UCSC browser) with arrows indicating the direction of transcription. The mammalian conservation track (UCSC genome browser) shows degree of placental mammal base pair conservation (20 species) and sequence conservation. (B) Represents lncRNA 441, which is located in antisense orientation to Per2 and only present in the Mus genus. (C) Shows the rat-specific lncRNA 6503, which is located in antisense orientation to Adcy1. (D) Transcript abundance of selected intragenic antisense lncRNAs in different adult Mmus tissues was validated by strand-specific quantitative RT-PCR. Each heatmap row represents one lncRNA. Areas are shaded according to abundance in per cent (white: 0 to black: 100%). (E) A three-way VENN diagram representing the intersect between the intragenic antisense lncRNA genes identified in each of the three rodents used in this study. Areas are shaded according to number of lncRNA genes (white: low to black: high).Validation of intragenic antisense ncRNA transcripts in rodents. (TIF) [file pgen.1002841.s002.tif]

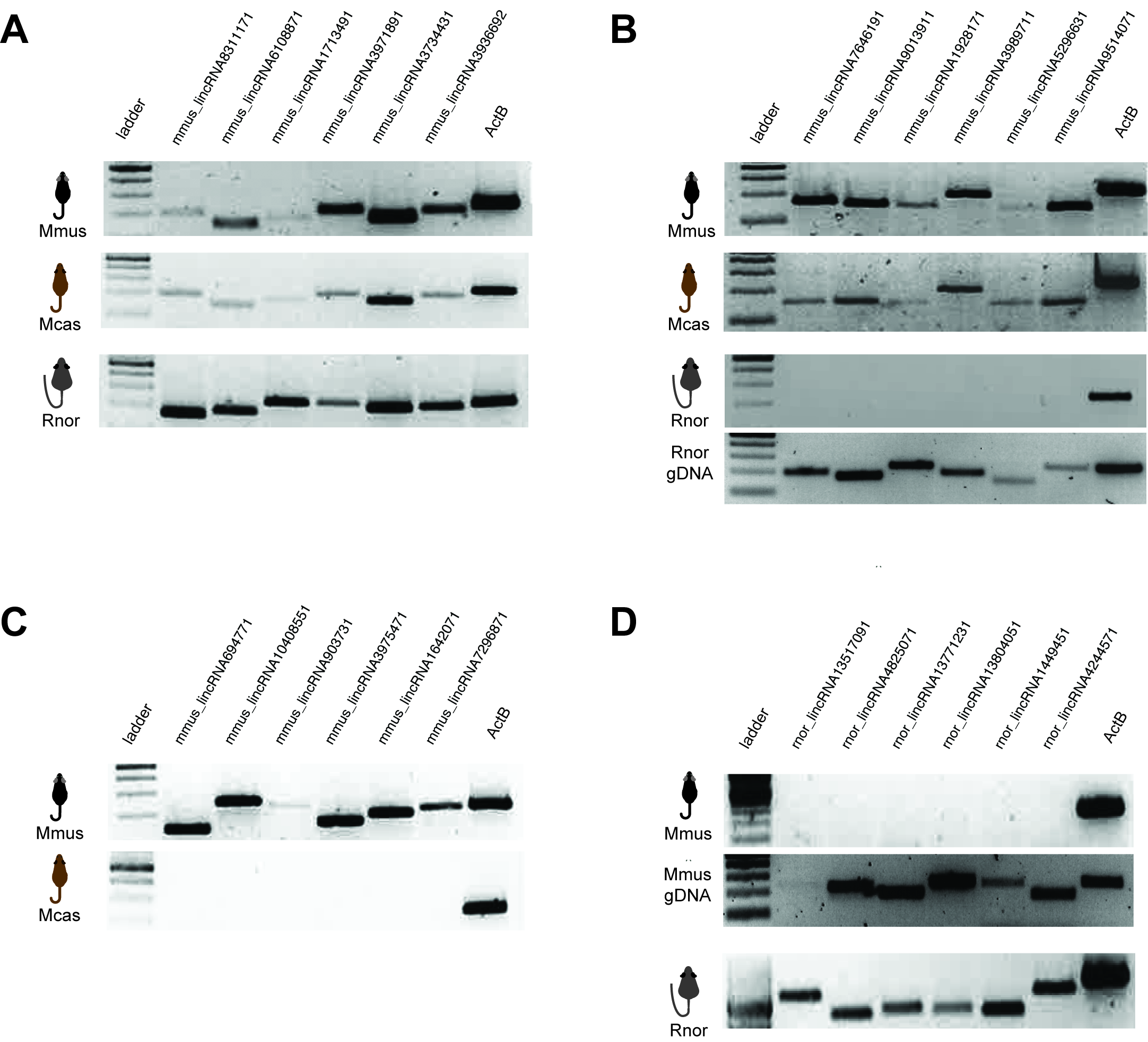

Supplement: Figure S3 — Validation of intergenic lncRNA expression in rodents. Liver expression of selected intergenic lncRNAs in Mmus, Mcas, and Rnor was tested by RT-PCR amplification: (A) Mmus, Mcas, and Rnor conserved intergenic lncRNAs, (B) Mus-genus specific intergenic lncRNAs, (C) Mmus-specific intergenic lncRNAs and (D) Rnor-specific intergenic lncRNAs. Actin B (ActB) expression in the three species was used as RT-PCR control. Genomic DNA (gDNA) of Rnor (B) and Mmus (D) was used for validating RT-PCR result. (TIF) [file pgen.1002841.s003.tif]

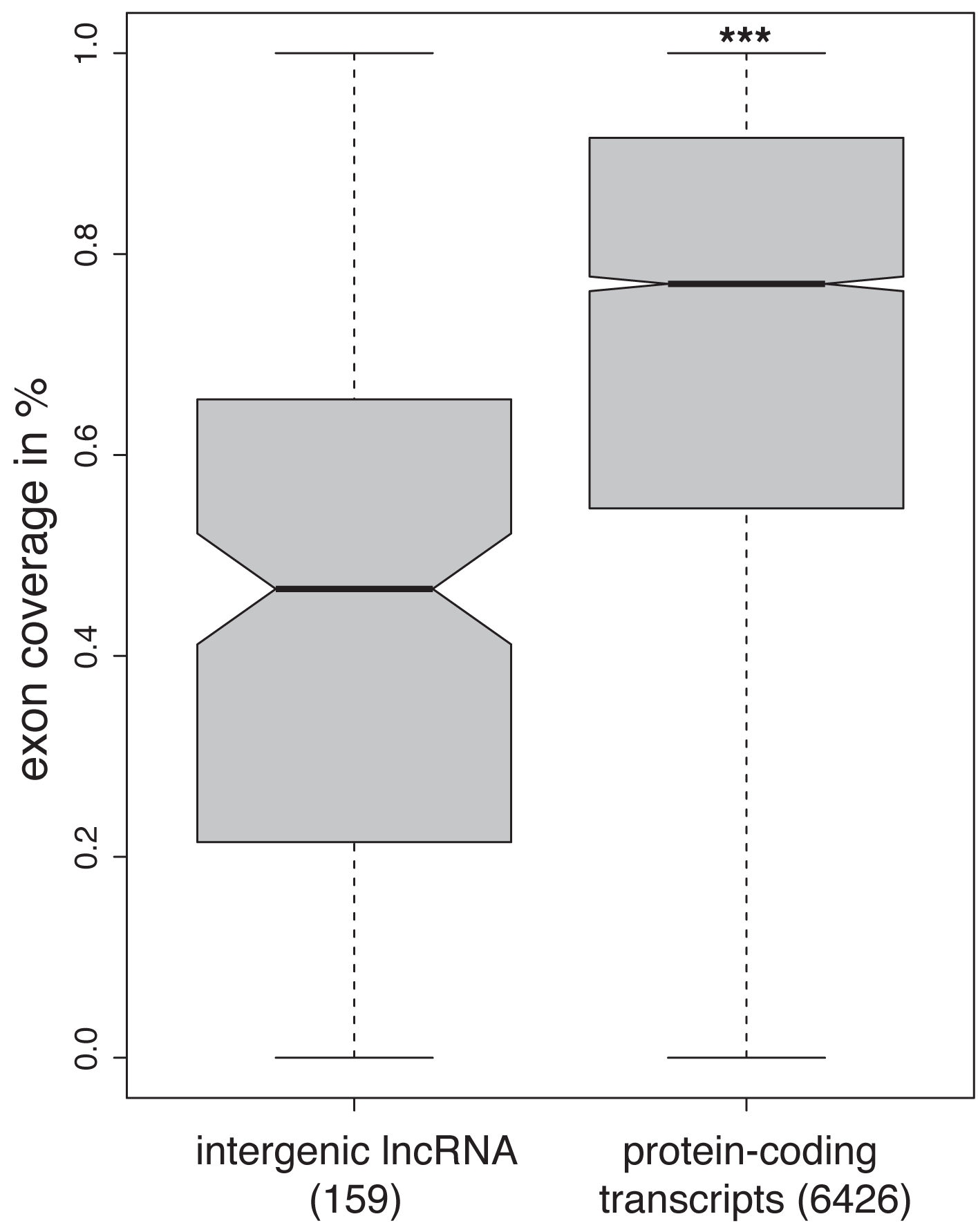

Supplement: Figure S4 — RNAseq read coverage is significantly higher accross protein-coding exons compared to intergenic lncRNAs. Coverage of rat RNAseq reads on rodent conserved intergenic lncRNA exons and protein-coding exons was determined. Exons of protein-coding transcrips have significantly higher coverage than those of intergenic lncRNAs (as indicated by asterisks ***, p<0.001). In parentheses are the numbers of intergenic lncRNA and protein-coding transcripts studied. (TIF) [file pgen.1002841.s004.tif]

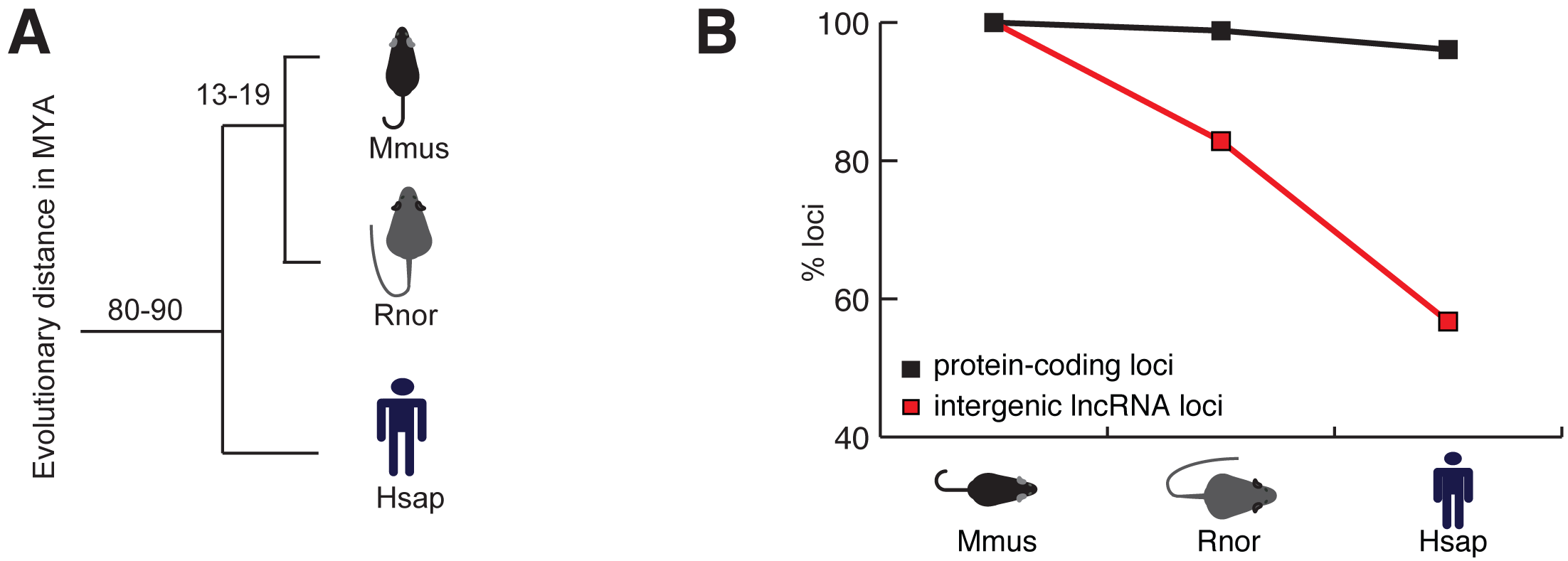

Supplement: Figure S5 — Transcriptional turnover of liver expressed intergenic lncRNA and protein-coding gene loci between rodents and human. (A) Phylogenetic tree representing the evolutionary relationship between human (Hsap), rat (Rnor) and mouse (Mmus). Humans and rodents shared a common ancestor about 80 to 90 million years ago (MYA). (B) Transcriptional turnover of liver-expressed Mmus protein-coding (black) and intergenic lncRNA loci (red) between rodents and human. (TIF) [file pgen.1002841.s005.tif]

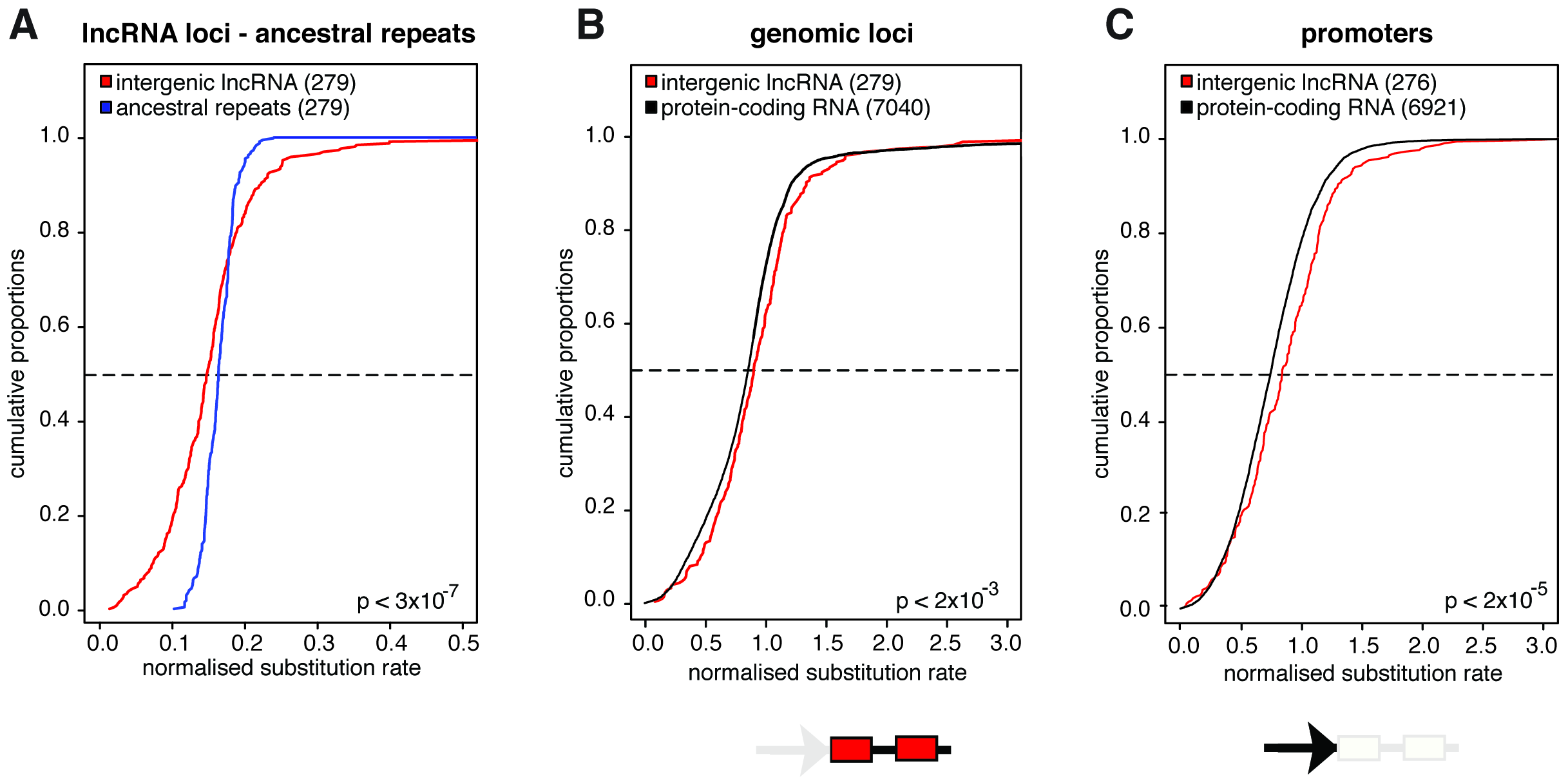

Supplement: Figure S6 — Nucleotide constraint between mouse and rat for promoter and transcribed loci of Mmus expressed intergenic lncRNAs and protein-coding genes. The cumulative distributions of substitution rates between mouse and rat for (A) 279 Mmus intergenic lncRNA loci (red) and neighbouring (<500 kb) ancestral repeat loci (AR, blue). Median substitution rate for intergenic lncRNA (dloci = 0.148) and AR (dAR = 0.164) indicates that intergenic lncRNA loci accumulated significantly fewer substitutions than ARs (two-tailed Mann-Whitney test, p<3×10^−7^). (B) 279 Mmus-expressed intergenic lncRNA loci (red) and 7040 Mmus protein-coding genes (black). Intergenic lncRNA loci (median dloci/dAR = 0.902) in comparison to protein-coding transcripts (median dloci/dAR = 0.857) accumulated substitutions at significantly higher rates (two-tailed Mann-Whitney test, p<2×10^−3^). (C) 276 Mmus intergenic lncRNA (red) and 6921 Mmus protein-coding proximal putative promoters (black). Putative proximal promoters are defined as the 400 bp upstream regions of the TSS. Between mouse and rat the proximal promoters of intergenic lncRNA evolved faster (median dpromoter/dAR = 0.843) than those of protein-coding genes (median dpromoter/dAR = 0.746) (two-tailed Mann-Whitney test, p<2×10^−5^). The substitution rate for each loci were normalised to the substitution rate measured for AR with matched G+C content in their vicinity (<500 kb). Numbers of loci studied are shown in parentheses. Black dashed line indicates 50% of the cumulative proportion. (TIF) [file pgen.1002841.s006.tif]

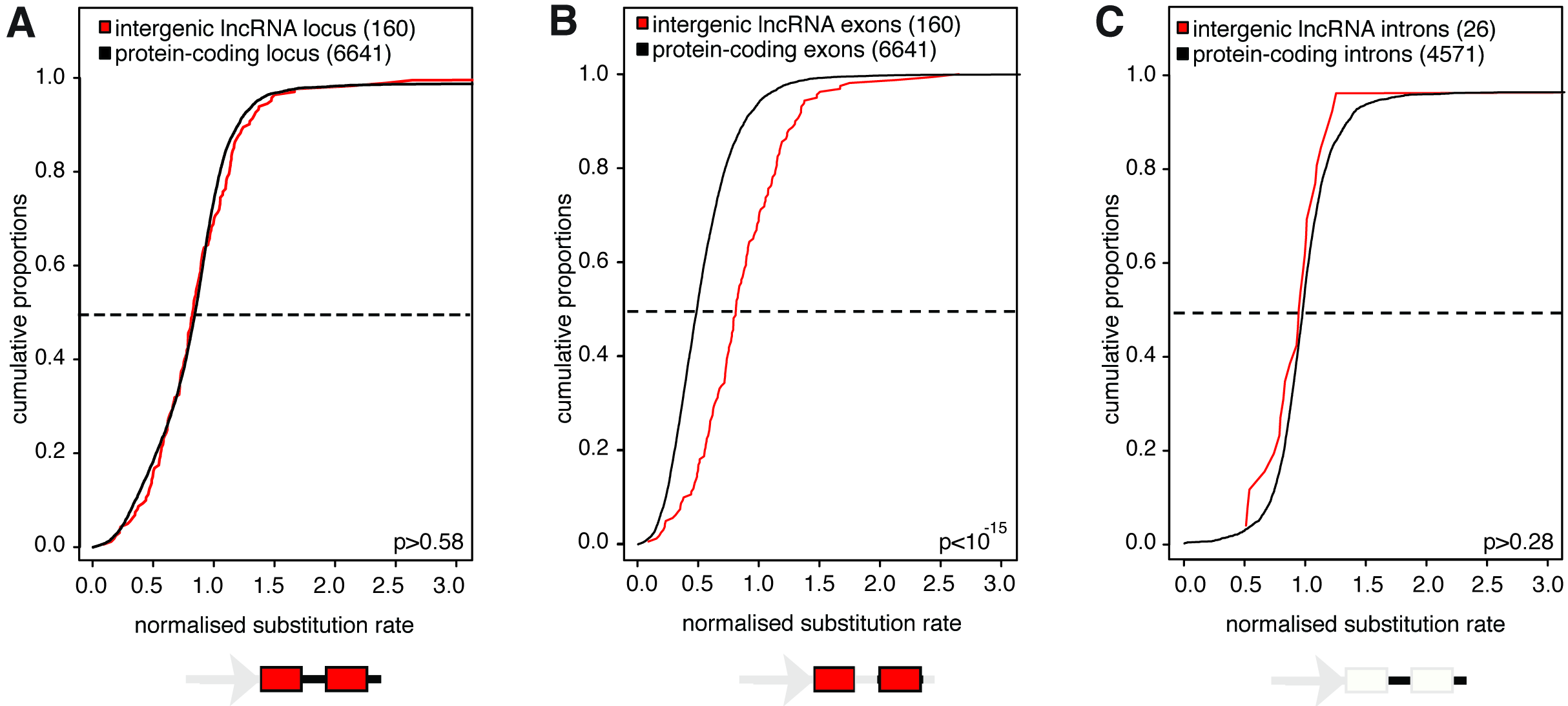

Supplement: Figure S7 — Nucleotide constraint between mouse and rat for different transcript features of rodent conserved intergenic lncRNA loci and protein-coding genes. The cumulative distributions of substitution rates between mouse and rat is shown for (A) 160 intergenic lncRNA loci and 6641 protein-coding gene loci whose expression is conserved between mouse and rat. Median substitution rates for intergenic lncRNA loci (dloci/dAR = 0.827) and protein-coding genes (dloci/dAR = 0.842) are not significantly different (two-tailed Mann-Whitney test, p>0.58). (B) Cumulative distribution of substitution rates for the exonic sequence of 160 intergenic lncRNA loci and 6641 protein-coding loci whose expression is rodent conserved. Intergenic lncRNA exons evolved under significantly (Mann-Whitney test, p<10−15) less constraint (median dexon/dAR = 0.805) than those of protein-coding genes (median dexon/dAR = 0.484). (C) Cumulative distribution of substitution rates for the intronic sequence of 26 intergenic lncRNA loci and 4571 protein-coding genes. Intergenic lncRNA introns (median dintron/dAR = 0.959) accumulated substitutions at similar rates to introns in protein-coding genes (median dintron/dAR = 0.986) (two-tailed Mann-Whitney test, p>0.28). The substitution rate for each loci were normalised to the substitution rate measured for ancestral repeats (AR) with similar G+C content in their vicinity (<500 kb). Cumulative proportion plots for intergenic lncRNAs and protein-coding genes are represented in red and in black, respectively. Number of loci studied are shown in parentheses. Black dashed line indicates 50% of the cumulative proportion. (TIF) [file pgen.1002841.s007.tif]

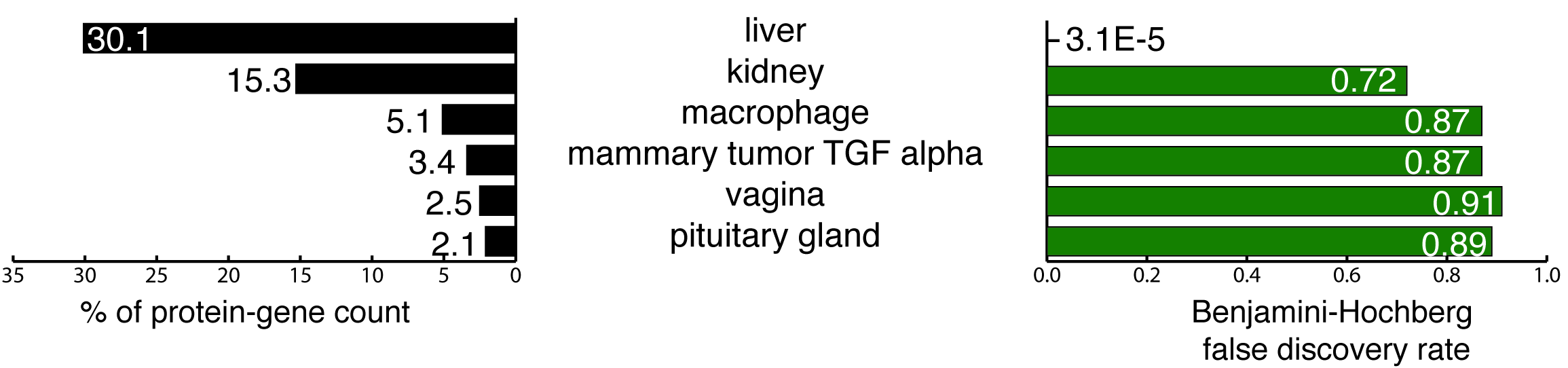

Supplement: Figure S8 — Expressed protein-coding genes located near intergenic lncRNAs hold liver-associated functional annotation. Classification of functional annotation (tissue) of protein-coding genes near intergenic lncRNAs. Left: percentage of protein-coding genes with assigned functional annotation (black bars), middle: functional annotation and right: false discovery rate (green bars). Each row represents one functional annotation (tissue). (TIF) [file pgen.1002841.s008.tif]

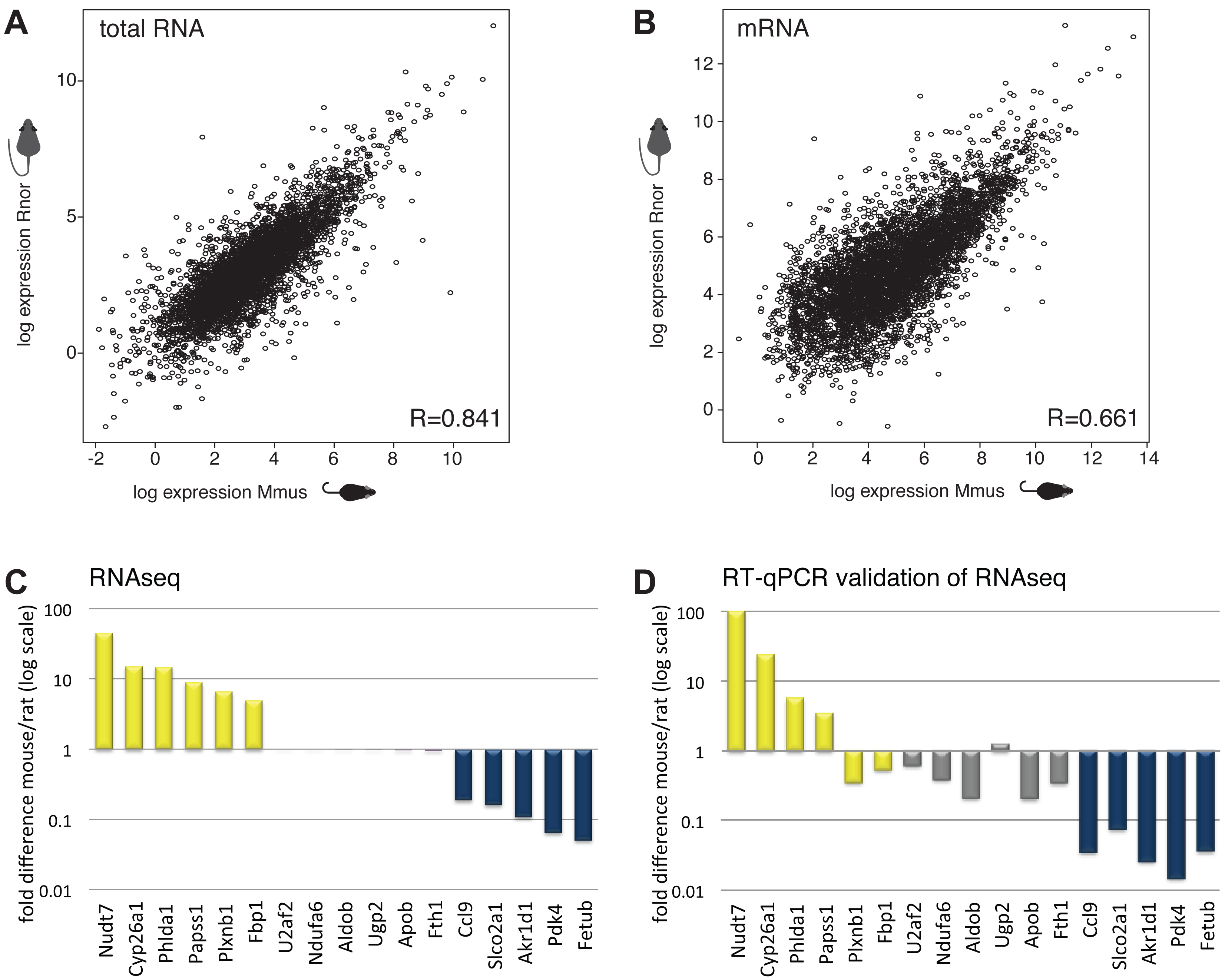

Supplement: Figure S9 — Normalised expression values of Mmus and Rnor one-to-one orthologous protein-coding genes correlate. Normalised expression level estimates [log(FPKM)] based on (A) total RNA and (B) mRNA sequencing reads of mouse (x-axis) and rat (y-axis) are positively correlated. Pearson correlation (R) are reported at bottom right of each panel. Median fold differences (log scale) of selected protein-coding gene products obtained from (C) RNAseq experiments were validated by (D) RT-qPCR of three independent biological replicates. The order of transcripts selected in (C) was maintained in (D). Yellow: mRNAs upregulated in mouse (or downregulated in rat), grey: transcripts with similar expression fold changes in mouse and rat, and blue: mRNAs downregulated in mouse (or upregulated in rat). (TIF) [file pgen.1002841.s009.tif]

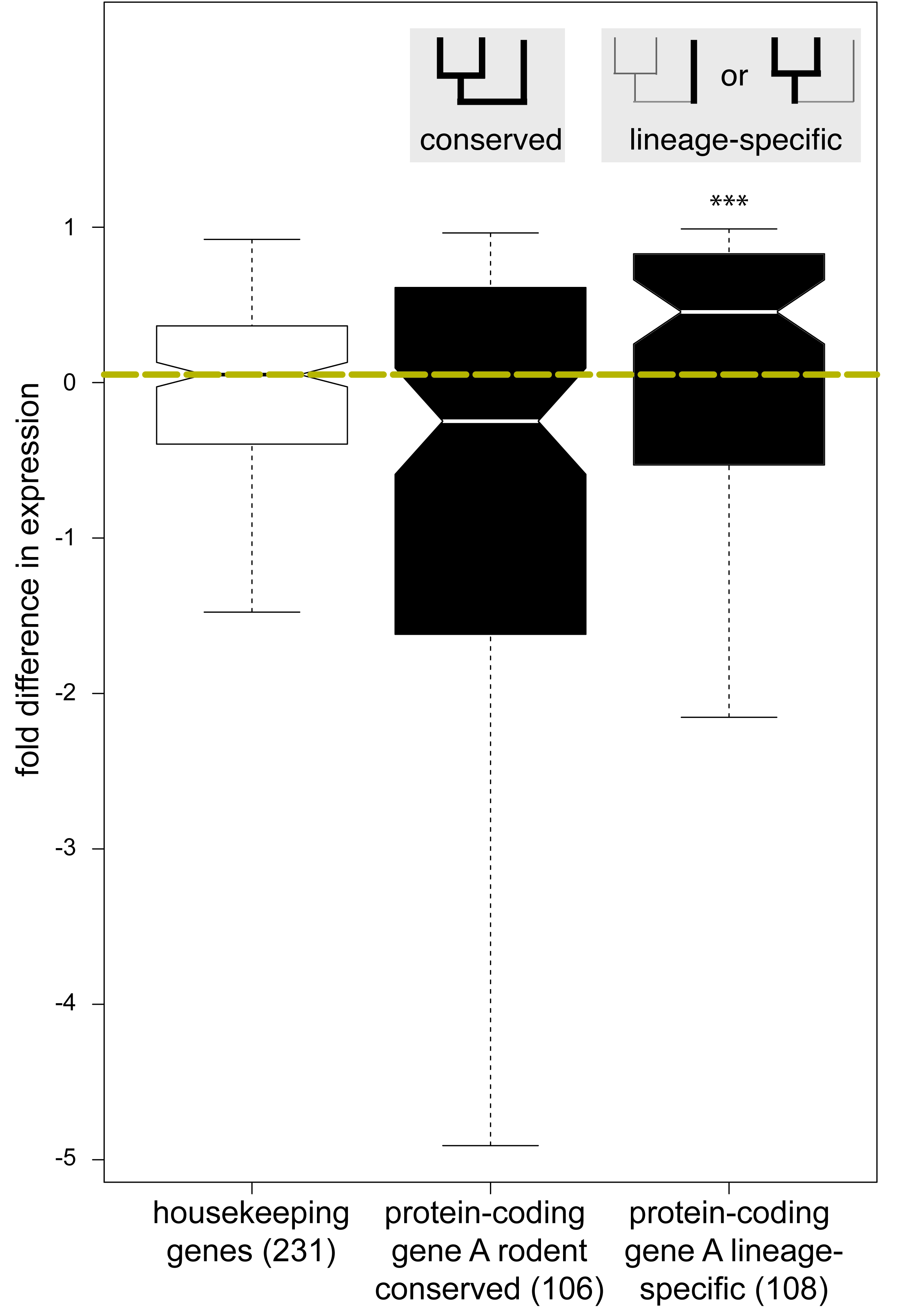

Supplement: Figure S10 — Lineage-specific intergenic lncRNAs are associated with increased expression of genomically adjacent protein-coding genes. mRNAseq based fold-difference in expression for one-to-one orthologous closest protein-coding gene to rodent conserved (conserved in Mmus, Mcas and Rnor) and lineage-specific (Mus genus- or Rnor-specific) expressed intergenic lncRNAs. Rodent conserved intergenic lncRNA gene expression is not associated with increased expression level (median fold-difference in expression = −0.248) of neighbouring protein-coding gene (housekeeping genes median fold difference in expression = 0.051, two-tailed Mann-Whitney test, p>0.18). In contrast lineage specific intergenic lncRNA expression is associated with significantly (2-tailed Mann-Whitney test, p<7×10^−5^, represented by asterisks [***]) increased expression of their neighbouring protein-coding genes (median fold difference in expression = 0.455). Yellow dashed line represents median fold-difference in expression between 231 Mmus and Rnor housekeeping genes. Numbers of loci studied are shown in parentheses. Normalised expression values were obtained from mRNAseq experiments [18]. (TIF) [file pgen.1002841.s010.tif]

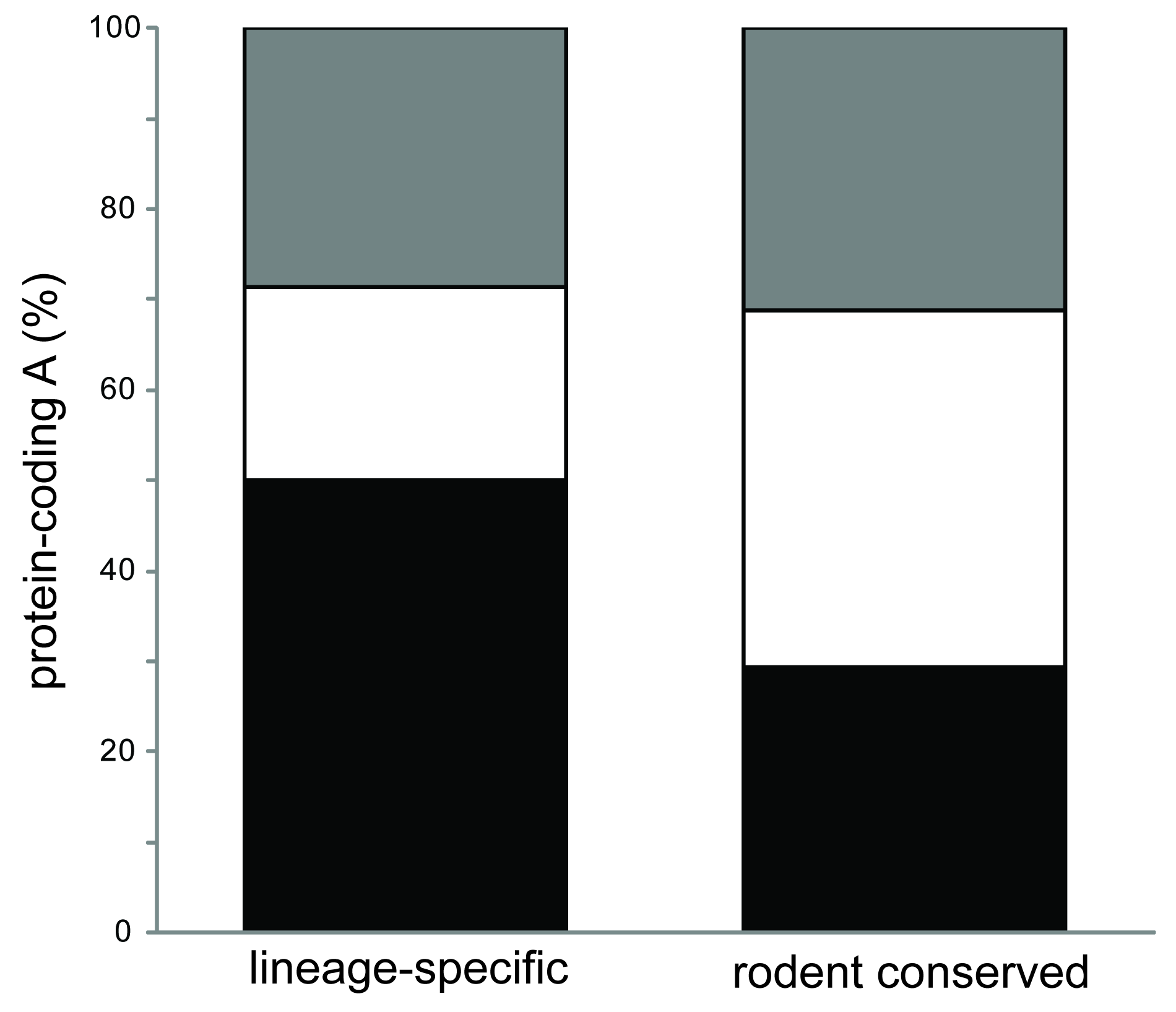

Supplement: Figure S11 — Lineage specific intergenic lncRNA transcription associates with consistent increased expression levels of neighbouring protein-coding genes. Expression levels for most protein-coding genes near lineage-specific intergenic lncRNAs (Mus genus- or Rnor-specific, left) are increased in comparison to protein-coding genes near rodent conserved intergenic lncRNAs (right). White: decrease, grey: ambiguous (inconsistent direction of change between two experiments), black: increase. (TIF) [file pgen.1002841.s011.tif]

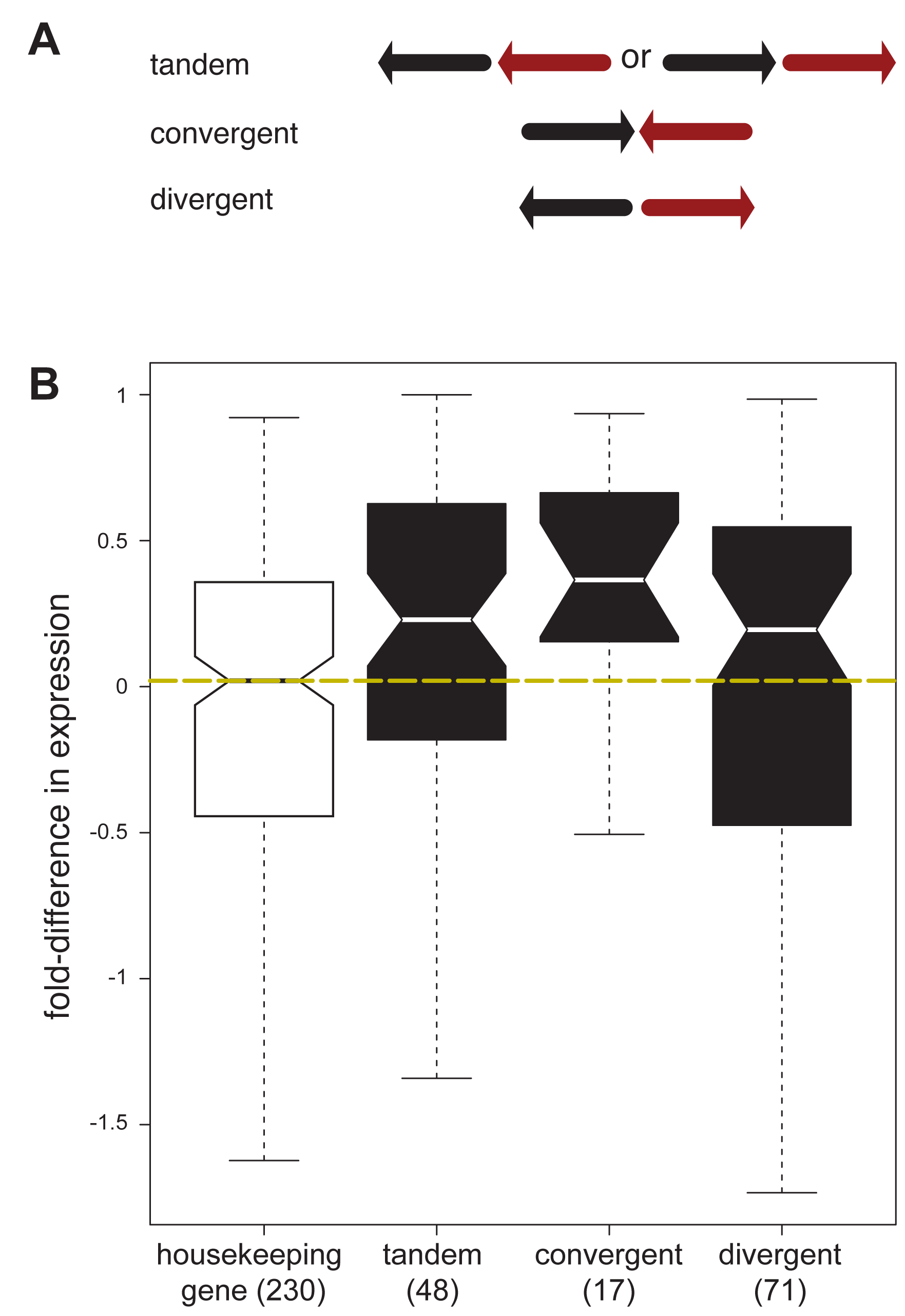

Supplement: Figure S12 — Lineage-specific intergenic lncRNAs are associated with increased expression of genomically adjacent protein-coding genes independent of relative orientations. (A) Relative orientations of lineage-specific intergenic lncRNAs (red) and their closest protein-coding neighbouring genes (black) are illustrated. Intergenic lncRNAs are placed downstream of its protein-coding loci in this diagram for illustrative purposes. Intergenic lncRNA and protein-coding gene pairs were divided into three classes: (i) tandem if transcription occurs in the same directions (48 pairs); (ii) convergent (17 pairs); and (iii) divergent (71 pairs). (B) Fold-difference in expression for one-to-one orthologous protein-coding gene pairs adjacent to lineage-specific (Mus genus- or Rnor-specific) intergenic lncRNA loci. The fold-difference in expression for Mmus and Rnor protein-coding genes was higher (two-tailed Mann-Whitney test; tandem: p-value<0.05; convergent: p<0.05; divergent: p<0.1) than the expected variation in expression based on 230 housekeeping genes (white). Yellow dashed line represents median fold-difference in expression between Mmus and Rnor housekeeping genes. In parentheses are the numbers of protein-coding genes studied. (TIF) [file pgen.1002841.s012.tif]

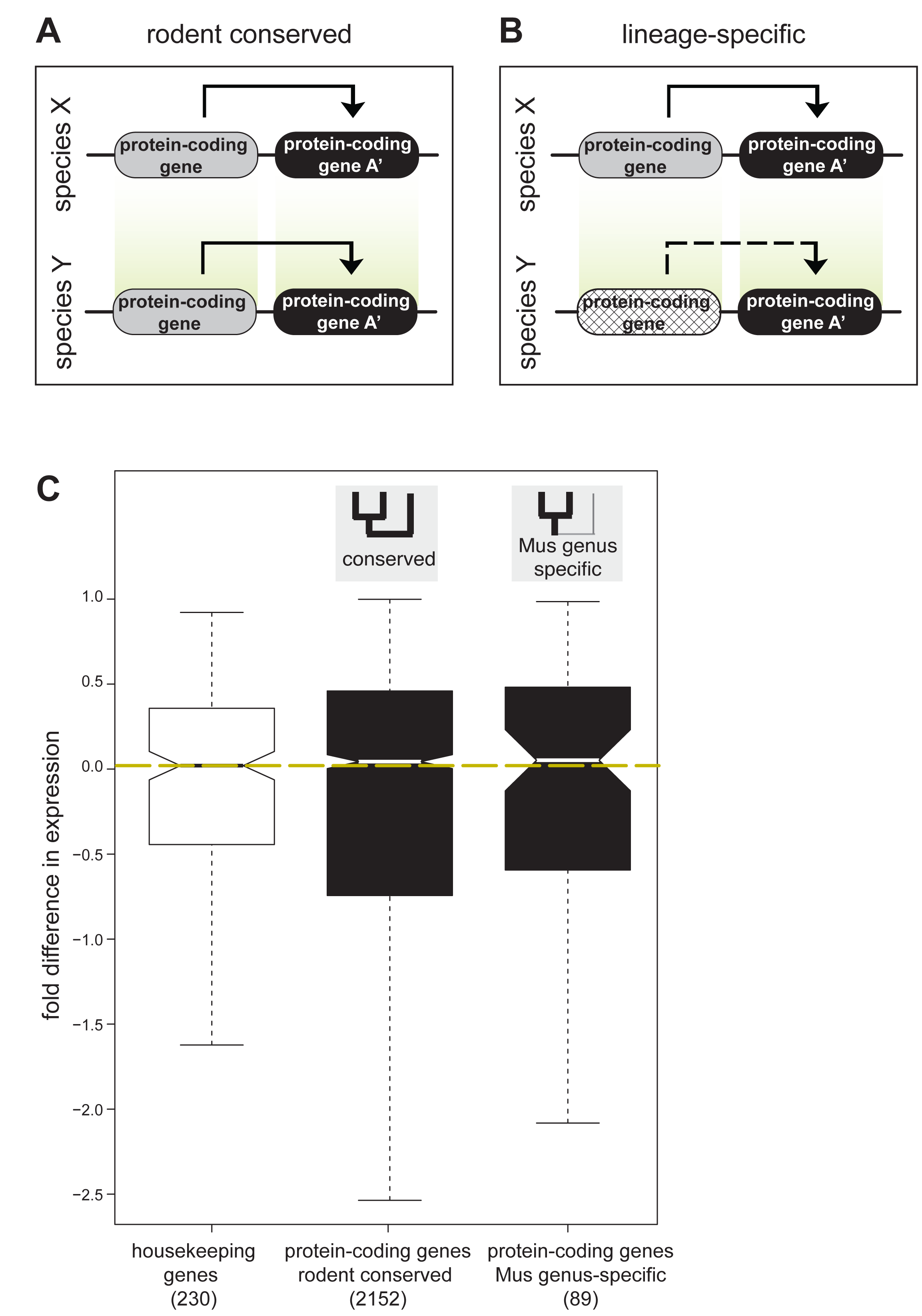

Supplement: Figure S13 — Rodent conserved and lineage-specific protein-coding genes are not associated with elevated expression of their closest neighbouring protein-coding gene. Effect of protein-coding gene (grey) transcription on their closest protein-coding genes A′ (black) was determined for (A) rodent conserved and (B) lineage-specific protein-coding gene pairs. (C) The median fold-difference in expression level between mouse and rat for protein-coding genes A′ closest to protein-coding genes with conserved expression in rodents (median fold-difference = 0.04) is not significantly different from the median fold-difference in expression for 230 housekeeping genes (two-tailed Mann-Whitney test, p>0.8). Lineage-specifically expressed protein-coding genes (Mus genus) are also not associated with significant (two-tailed Mann-Whitney test, p>0.4) differences in expression of their closest protein-coding gene (median fold-difference = 0.05). The yellow dashed line represents median fold-difference in expression between Mmus and Rnor housekeeping genes. Numbers of protein-coding genes studied are shown in parentheses. (TIF) [file pgen.1002841.s013.tif]

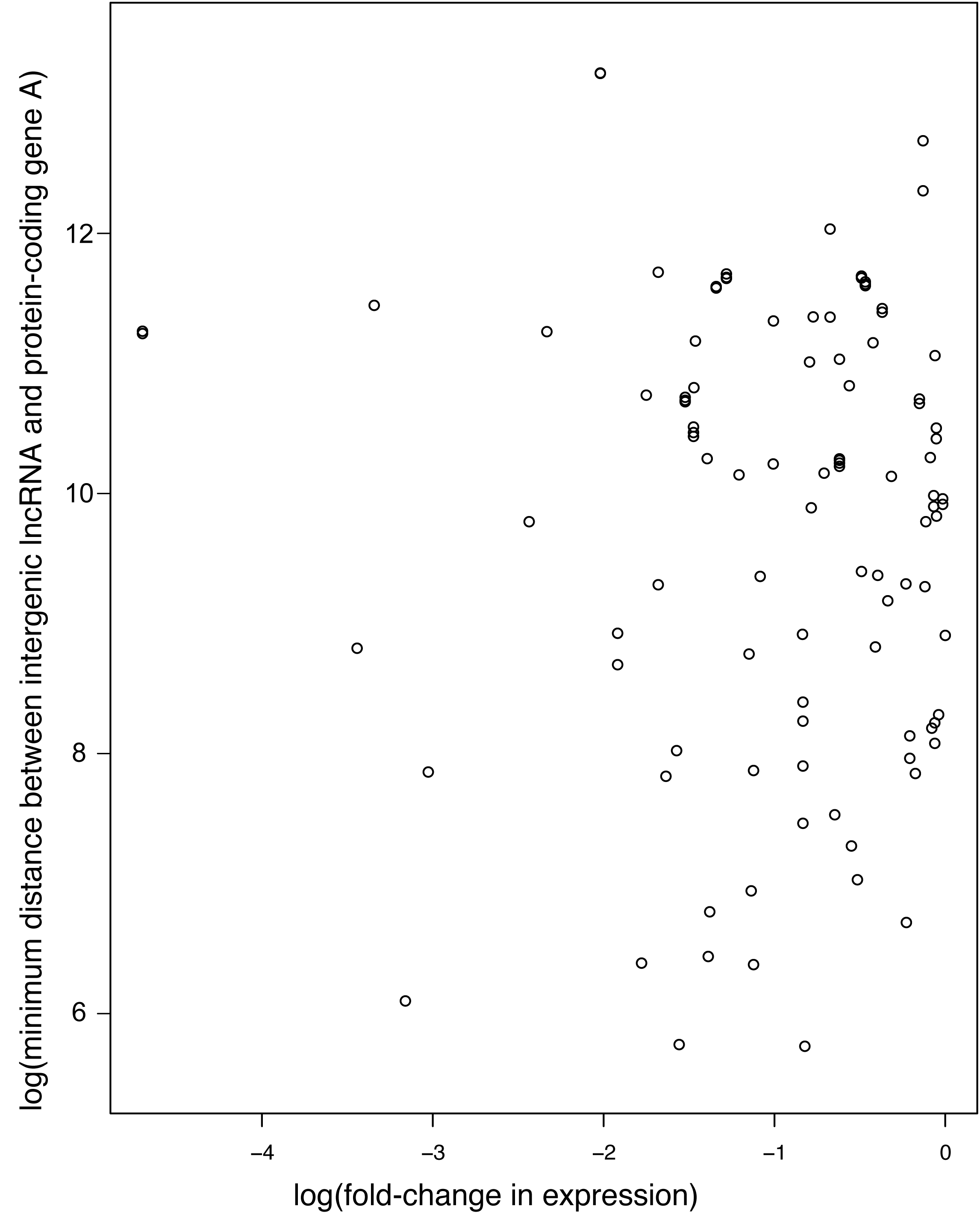

Supplement: Figure S14 — Distance of protein-coding genes to nearest lineage–specific intergenic lncRNA loci and expression levels of protein-coding genes do not correlate. The lineage-specific effect of intergenic lncRNA expression on its neighbouring protein-coding genes does not correlate with the distance between the two loci. The smallest distance (base pairs) between the TSSs for each intergenic lncRNA and protein-coding gene A pair (y-axis, log-scale) is plotted against the corresponding fold-difference in expression between Mmus and Rnor (x-axis, log-scale). These measures are not significantly (p = 0.76) correlated (Pearson correlation coefficient R = −0.026). (TIF) [file pgen.1002841.s014.tif]
